# Comparative transcriptome analysis to identify putative genes involved in thymol biosynthesis pathway in medicinal plant *Trachyspermum ammi* L.

**DOI:** 10.1038/s41598-018-31618-9

**Published:** 2018-09-07

**Authors:** Mehdi Soltani Howyzeh, Seyed Ahmad Sadat Noori, Vahid Shariati J., Mahboubeh Amiripour

**Affiliations:** 1 Department of Agronomy and Plant Breeding Sciences, College of Abouraihan, University of Tehran, Tehran, Iran; 2 Molecular Biotechnology Department, National Institute of Genetic Engineering and Biotechnology, Tehran, Iran; 3 NIGEB Genome Center, National Institute of Genetic Engineering and Biotechnology, Tehran, Iran

## Abstract

Thymol, as a dietary monoterpene, is a phenol derivative of cymene, which is the major component of the essential oil of *Trachyspermum ammi* (L.). It shows multiple biological activities: antifungal, antibacterial, antivirus and anti-inflammatory. *T. ammi*, commonly known as ajowan, belongs to Apiaceae and is an important medicinal seed spice. To identify the putative genes involved in thymol and other monoterpene biosynthesis, we provided transcriptomes of four inflorescence tissues of two ajowan ecotypes, containing different thymol yield. This study has detected the genes encoding enzymes for the go-between stages of the terpenoid biosynthesis pathways. A large number of unigenes, differentially expressed between four inflorescence tissues of two ajowan ecotypes, was revealed by a transcriptome analysis. Furthermore, differentially expressed unigenes encoding dehydrogenases, transcription factors, and cytochrome P450s, which might be associated with terpenoid diversity in *T. ammi*, were identified. The sequencing data obtained in this study formed a valuable repository of genetic information for an understanding of the formation of the main constituents of ajowan essential oil and functional analysis of thymol-specific genes. Comparative transcriptome analysis led to the development of new resources for a functional breeding of ajowan.

## Introduction

Terpenoids are the biggest group of plant secondary metabolites, and interest in isolated terpenoids has been growing in recent years due to their pharmaceutical or pharmacological utility. They are the main components of many essential oils extensively used as fragrances, flavoring, scenting agents, active ingredients, and intermediates in cosmetics, food additives, and synthesis of perfume chemicals^1^. Thymol is a monoterpene compound derived from isoprene hydrocarbure (2-methyl-1, 3-butadiene) and formed by the attachment of two or more isoprene molecules. Thymol shows multiple biological activities: antifungal^2^, antibacterial^3^, antiviral^4^, anti-inflammatory^5^, antioxidant^6^, free radical scavenging^7^ and anti-lipid peroxidative^8^ properties. Most of the monoterpenes are produced by the modification of GPP, the initial parent compound^9^. Thus, monoterpene biosynthesis can be indicated in four phases: (1) generation of the terpenoid building units (IPP and DMAPP); (2) creation of GPP by condensation of IPP and DMAPP using prenyltransferase; (3) transformation of GPP into the monoterpene parent skeleton; and (4) conversion of the parent structure to various formations. Catabolism and evaporative losses of plant monoterpenes appeared to play minor roles in influencing the production yields of these natural products^10^.

Regarding the biosynthetic pathway of terpenoid compounds, terpene synthases (TPS) used several steps of cyclization and oxidation to catalyze the precursors of each terpenoid families and, as the key enzymes, formed a simple or mixed compound of reaction products of terpenoid metabolites^11,12^. In addition to the existence of a conserved motif (DDxxD), which is involved in binding metal ion co-factors, the species relationships dominate the similarity of TPS sequences regardless the specificity of the substrate or the end-product^13^. The specificity of a reaction product for a terpene synthase changed by a single amino acid replacement, may lead to qualitative variations in volatile profiles^14,15^. Further modifications of the terpene products are also made by other enzymes such as dehydrogenases and cytochrome P450 mono-oxygenases^16,17^.

Ajowan (*Trachyspermum ammi*), as a family member of Apiaceae, is an essential oil, annual, and highly valued medicinally important seed spice. Seeds of *T. ammi* are a rich repository of secondary metabolites used as a traditional drug and food in some countries such as India and Iran. Ajowan seeds include high yields of essential oils with valuable main monoterpenes such as thymol^18^. *T. ammi* has carminative, stomachic, sedative, antibacterial, antifungal, and anti-inflammatory effects. *T. ammi* oils and their constituents are largely employed for the preparation of tooth paste, cough syrup, and pharmaceutical and food flavoring^19^. *T. ammi* essential oil is a blend mainly of monoterpenes. The major components were reported as thymol, γ-terpinene, and p-cymene^20,21^, which make up approximately 98% of the oil. Secondary metabolite biosynthesis and accumulation depend on the enzymes and genes having tissue-specific expression patterns^22^.

Medicinal plant taxa cover a wide range of plants, which produce various classes of natural products but mostly have limited genomic or transcriptomic resources. Novel genes from these non-model species can be detect by the next generation sequencing (NGS) techniques as valuable genomic tools, which have been used for identifying and characterizing secondary metabolism genes and their pathways^23^. Many plant genomes have been sequenced since the development of NGS technology, including plants such as wheat^24^, barley^25^, soybean^26^ and others, providing huge quantities of data to explain the complex biosystem of plant species. RNAseq, as a revolutionary tool, uses the deep-sequencing technology for transcriptome profiling. It can be used for different goals, such as transcriptome quantification, differential expression, transcript annotation, novel transcript identification, molecular marker development, alternative splicing^27–29^, and polymorphism detection at the transcriptome level^30,31^. This technique, as an efficient, cost-effective, and high-throughput technology, can be performed for plant species with or without a genome reference, making it a suitable alternative for analyzing non-model plant species without any genomic sequence source^32,33^. Several non-model medicinal plants such as the *Allium tuberosum, Papaver somniferum*, *Gentiana rigescens*, and *Phyllanthus amarus* have been studied with the help of transcriptome sequencing^34–37^, which has helped acquire more knowledge of secondary metabolites biosynthesis in these plants. In this study, the differential gene expressions of inflorescence tissues of two distinct *T. ammi* ecotypes, containing different amounts of oil content and thymol yield among 23 indigenous ecotypes, gathered from various parts of Iran^21^, were studied using a transcript pair-end sequencing strategy. The *de novo* assembly was performed by an Illumina sequencing of the extracted RNA from four inflorescence tissues. The transcriptome was annotated and the pathways of thymol and other terpenoid were analyzed.

## Results

### Analysis of main metabolite components in different inflorescence tissues

**Figure 1:**
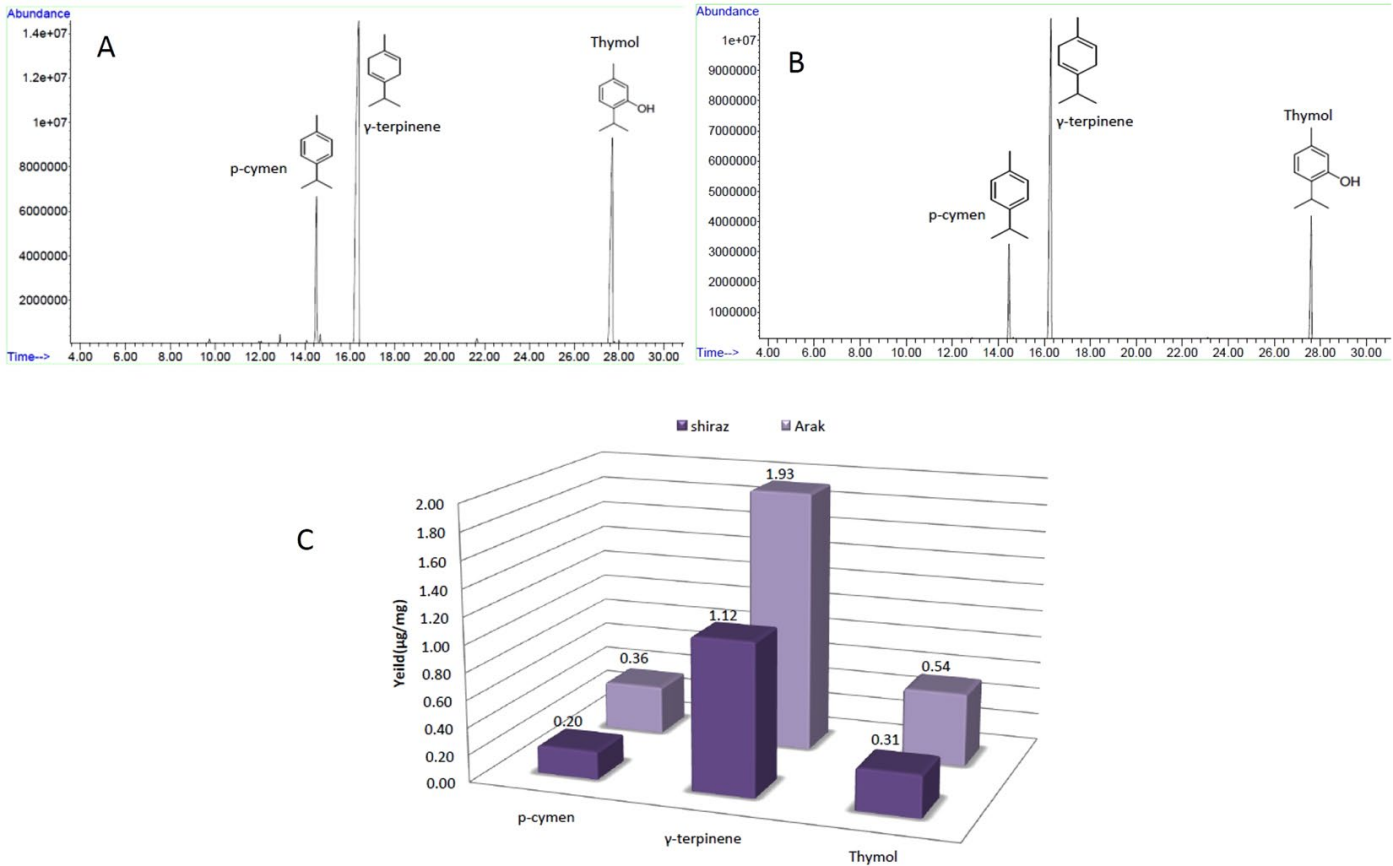
GC/MS profile of *Trachyspermum ammi* inflorescence essential oil. (**A**) Arak ecotype. (**B**) Shiraz ecotype. (**C**) Yield of essential oil’s main components.

Metabolite analysis of the inflorescence tissues of two ajowan ecotypes (Fig. 1) showed that thymol, as well as γ-terpinene and p-cymene, were the main monoterpenes in the inflorescence tissues of the Arak and Shiraz ecotypes (Fig. 1). The maximum thymol accumulated in the Arak ecotype (0.54 μg/mg inflorescence dry weight). The γ-terpinene and the p-cymene accumulation was also more in the inflorescence tissues of the Arak ecotype (Fig. 1C).

### Establishment of ajowan transcriptomes

**Figure 2:**
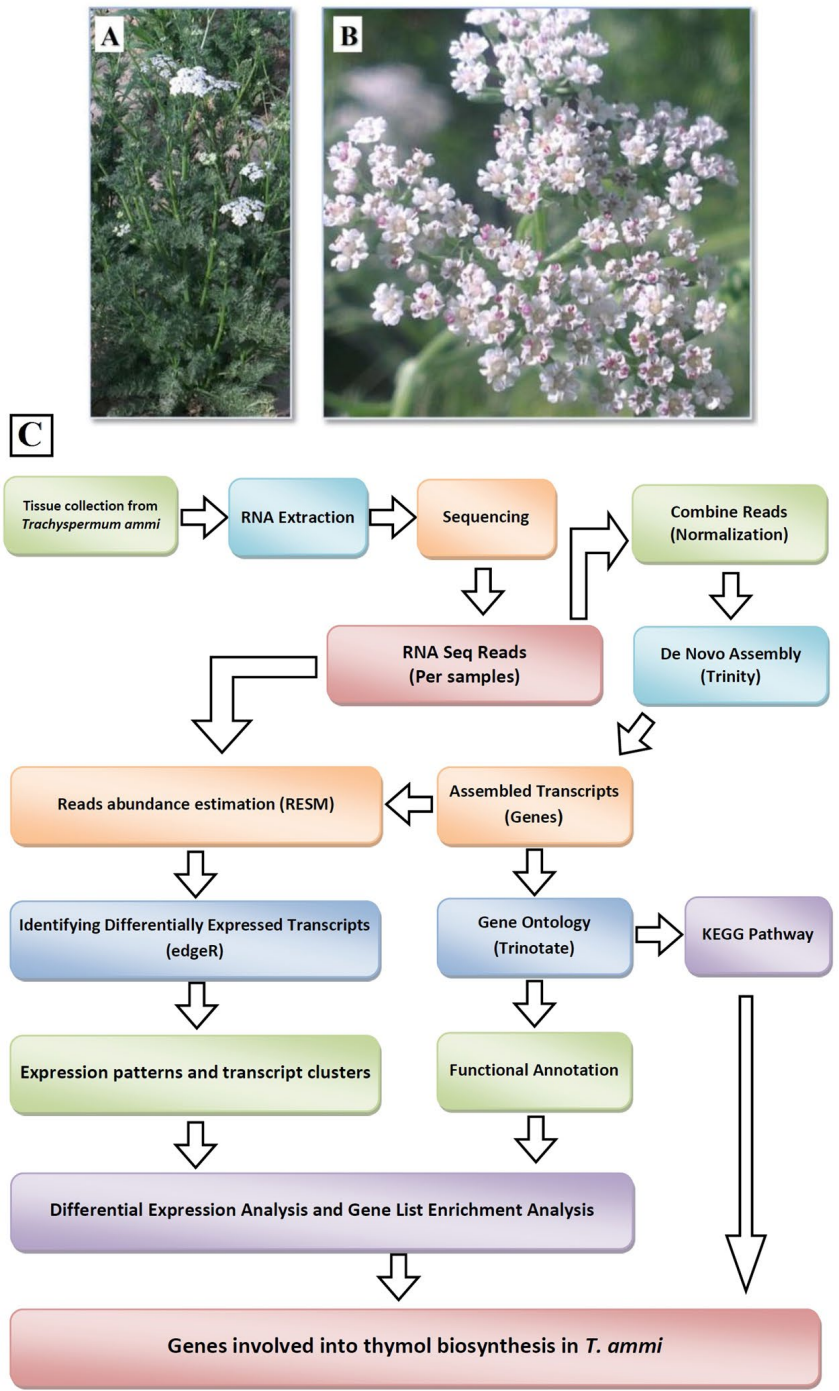
Plant materials and RNA-seq analysis workflow. (**A**) *T. ammi* plant. (**B**) Inflorescence tissue of *T. ammi*. (**C**) Schematic overview of *de novo* RNA-seq analysis workflow of inflorescences of *T. ammi*.

To study thymol biosynthesis, short-read transcriptome sequencing from the inflorescence tissues of two ecotypes (Arak and Shiraz) was carried out (Fig. 2). Sequencing runs of the inflorescence tissues of Arak-3 and Arak-10 yielded 43,056,120 and 42,260,830 of high-quality reads, respectively. Similarly, 51,965,127 and 47,891,026 high-quality reads were generated from the inflorescence tissues of Shiraz-17 & Shiraz-21, respectively. Details of the generated sequencing data are illustrated in the Supplementary Table S1.

### De novo assembly and annotation

To reconstruct the transcriptome dataset for ajowan, a total of four cDNA libraries were generated from the inflorescence tissues of the two ecotypes and were sequenced using the HiSeq. 2000 platform. A Trinity assembly of combined reads was selected for further analysis, which produced 123,488 unigenes with N50 of 994 bp and 151,115 transcripts with N50 of 1291 bp. The GC content of the assembled combined reads in the inflorescence tissues of two ecotypes was 38.35% (Table 1). The Trinity assembly of the combined reads obtained from Arak-3, Arak-10, Shiraz-17 and Shiraz-21 libraries resulted in the generation of 62,380, 68,051, 72,074 and 73,093 unigenes, respectively (Supplementary Table S1). The unigenes had an average length from 826 bases to 892 bases in all the libraries. Among all the unigenes, more than 27% were recognized as large unigenes, longer than 1000 bases in all libraries of the two ecotypes. The length distribution of the unigenes from all libraries is displayed in Supplementary Fig. S1.

**Table 1:** Trinity assembly stats report of assembled contigs.

Counts of transcripts, etc.		
Total trinity ‘genes’	Total trinity transcripts	Percent GC
123488	151115	38.35
**Trinity assembly stats**		
**Stats**	**based on all transcript contigs**	**based on only LONGEST ISOFORM per ‘GENE’**
Contig N10	3069	2894
Contig N20	2363	2189
Contig N30	1944	1740
Contig N40	1603	1346
Contig N50	1291	994
Median contig length	454	385
Average contig	782.02	659.33
Total assembled bases	118175363	81419608

**Figure 3:**
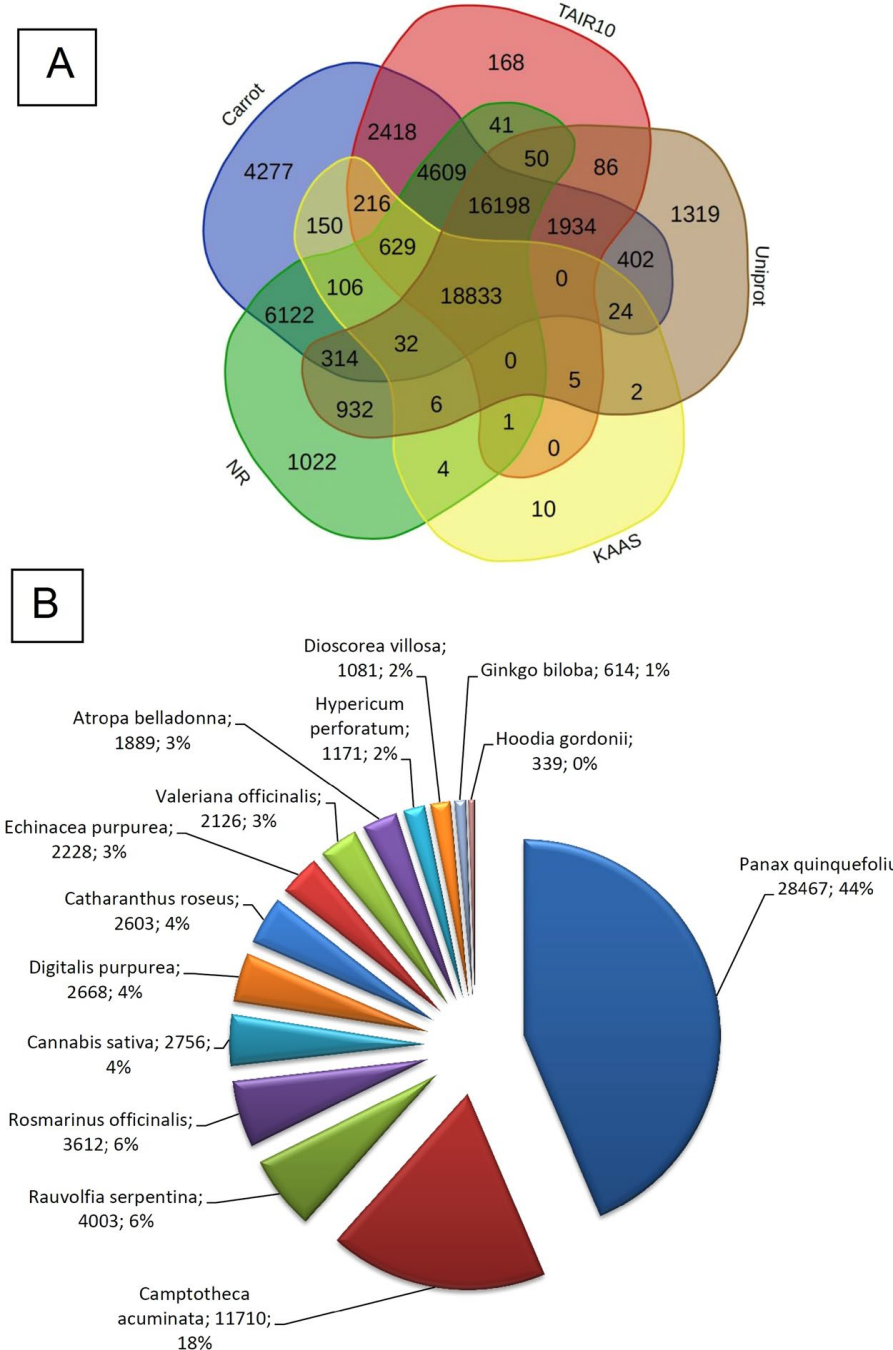
The BLAST annotation results of assembled unigenes. (**A**) Venn diagram of BLASTX results against different databases. (**B**) The BLASTX annotation results against the Medicinal Plant Genome Resources (MPGR) protein database.

The unigenes’ annotation was done using BLASTx against KAAS, TAIR10, Uniprot, NCBI protein (NR) and carrot genome databases (Supplementary Table S2). BLAST results indicated an extensive coverage of *T. ammi* transcriptomes. A total of 20,018 (16.2%), 40,137 (32.5%), 45,188 (36.5%), 48,899 (39.6%) and 56,264 (45.6%) unigenes from all the libraries were annotated against KAAS, Uniprot, TAIR10, NCBI protein (NR) and carrot genome database, respectively (Fig. 3A). The number of commonly annotated unigenes was 18,833 (Fig. 3A). Also BLASTx results against Medicinal Plant Genomics Resource (MPGR) protein database indicated that *Panax quinquefolius* had the most number of annotated unigenes (28467, 44%) with *T. ammi* assembled unigenes among other medicinal plants (Fig. 3B).

### Gene ontology classification

**Figure 4:**
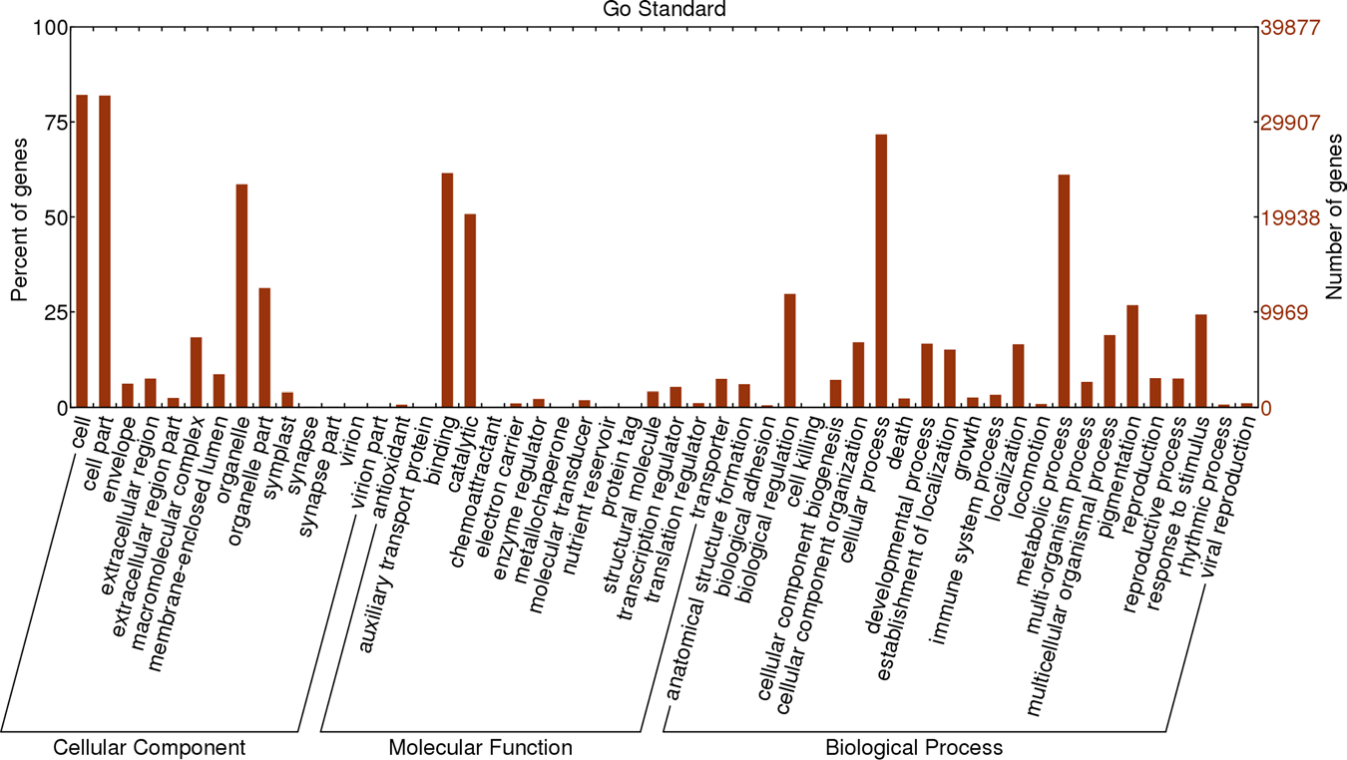
GO functional classification of assembled unigenes. Bars represent the percent and number of assignments of unigenes to each GO term.

In order to provide a functional classification of unigenes, GO assignments was created by Trinotate software and visualization of the Gene ontology classification was done by using Web Gene Ontology Annotation Plot (WEGO). The WEGO Plot showed that all unigenes were classified into 55 functional categories (Fig. 4). Among all the categories of cellular components (CC), molecular function (MF), and biological process (BP) of Gene Ontology Annotation, the dominant categories were ‘cell’ and ‘cell part’ (≥75%) (Fig. 4). Furthermore, ‘biological regulation’, ‘cellular process, ‘metabolic process’ and ‘response to stimulus’ categories had high percentages (Fig. 4). Out of the total categorized unigenes, 24,373 unigenes (61.1%) were assigned to the ‘metabolic process’ category, among which 1303 unigenes (3.3%) were assigned to the ‘secondary metabolic process’ category.

### Functional KEGG pathways identification

The functional biological pathways in *T. ammi* were identified by mapping 123,488 unigenes from the assembly to the canonical pathways reference in KEGG using KAAS. The result indicated that all unigenes were assigned to 355 KEGG pathways (Supplementary Table S3). In the secondary metabolic pathway (ko01110), 2,316 unigenes were identified, which related to 399 KEGG genes ko numbers (Table 2). Among all the secondary metabolic pathways, the ‘Phenylpropanoid biosynthesis [PATH: ko00940]’ cluster represented the largest group (242 members) followed by the ‘Terpenoid backbone biosynthesis [PATH: ko00900] (112 members) and ‘Carotenoid biosynthesis [PATH: ko00906]’ (76 members) clusters. ‘Monoterpenoid biosynthesis [PATH: ko00902]’ included 41 members (Table 3).

**Table 2:** Global and overview KEGG pathway maps of *Trachyspermum ammi* transcripts defined by KAAS.

KEGG Pathways	Reference KEGG Pathway Number (Ko)	N. of genes in each KEGG pathway	N. of identified genes in KEGG Pathway	Percent of identified genes in each KEGG Pathway	N. of unigenes for each KEGG Pathway	N. of unigenes per identified gene in map
Metabolic pathways	1100	2660	849	31.9	4374	5.2
Biosynthesis of secondary metabolites	1110	995	399	40.1	2316	5.8
Carbon metabolism	1200	342	92	26.9	685	7.4
2-Oxocarboxylic acid metabolism	1210	75	29	38.7	132	4.6
Fatty acid metabolism	1212	70	25	35.7	284	11.4
Biosynthesis of amino acids	1230	227	99	43.6	538	5.4

**Table 3:** Transcripts related to secondary metabolite biosynthesis in *T. ammi*.

category	KEGG Pathways	Reference KEGG Pathway Number (Ko)	N. of genes in each KEGG pathway	N. of identified genes in KEGG Pathway	Percent of identified genes in each KEGG Pathway	N. of unigenes for each KEGG Pathways	N. of unigenes per identified gene in map
Metabolism of terpenoids and polyketides	Terpenoid backbone biosynthesis	900	53	30	56.6	112	3.7
	Monoterpenoid biosynthesis	902	23	6	26.1	41	6.8
	Sesquiterpenoid and triterpenoid biosynthesis	909	66	9	13.6	44	4.9
	Diterpenoid biosynthesis	904	42	9	21.4	57	6.3
	Carotenoid biosynthesis	906	46	20	43.5	76	3.8
	Brassinosteroid biosynthesis	905	10	8	80.0	35	4.4
	Zeatin biosynthesis	908	8	6	75.0	82	13.7
Biosynthesis of other secondary metabolites	Phenylpropanoid biosynthesis	940	33	18	54.5	242	13.4
	Stilbenoid, diarylheptanoid and gingerol biosynthesis	945	13	5	38.5	61	12.2
	Flavonoid biosynthesis	941	19	12	63.2	45	3.8
	Flavone and flavonol biosynthesis	944	12	3	25.0	7	2.3
	Anthocyanin biosynthesis	942	14	4	28.6	36	9.0
	Isoflavonoid biosynthesis	943	13	2	15.4	3	1.5
	Indole alkaloid biosynthesis	901	10	2	20.0	5	2.5
	Isoquinoline alkaloid biosynthesis	950	42	9	21.4	63	7.0
	Tropane, piperidine and pyridine alkaloid biosynthesis	960	26	8	30.8	58	7.3
	Caffeine metabolism	232	9	3	33.3	17	5.7
	Betalain biosynthesis	965	7	1	14.3	2	2.0
	Glucosinolate biosynthesis	966	15	2	13.3	17	8.5

### Identification and classification of differentially expressed genes

**Figure 5:**
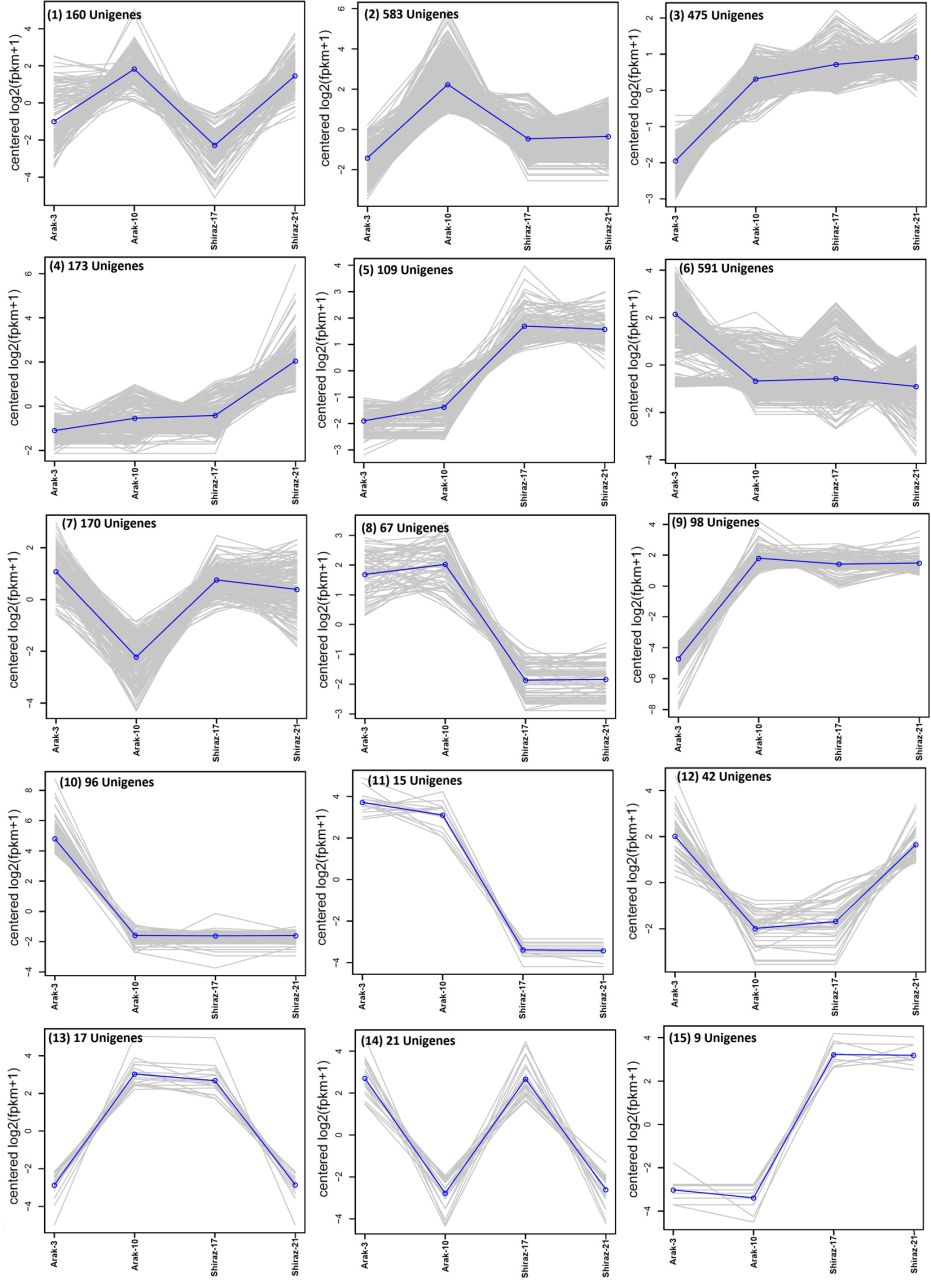
Expression pattern clustering of unigenes. Differentially expressed unigenes (Fold change ≥ 4 and p_value ≤ 1e-3) in four different genotypes of inflorescence tissues of two ajowan ecotypes. Blue line in each cluster shows common expression pattern of all the unigenes of the cluster.

Identification of differentially expressed genes was done based on fold change >4 and p_value < 1e-3. Out of 123,488 unigenes obtained from the Trinity assembly, 2,626 unigenes were recognized as differentially expressed unigenes in four inflorescence tissues of two ecotypes. Out of the 2,626 differentially expressed unigenes, 2,091 were annotated using different databases (Supplementary Table S2). The differentially expressed unigenes, which were uniquely annotated against KAAS, Uniprot, NR, TAIR10 and carrot genome databases were 866, 1,539, 1,773, 1,705 and 2,010, respectively (Supplementary Table S2 and Fig. S2). The clustering of expression patterns of the differentially expressed unigenes in inflorescence tissues of four genotypes is showed in Fig. 5. Among all the 15 clusters, Clusters 8 and 11 represented unigenes that were over expressed in high oil content ecotype tissues (Arak-3 & Arak-10) compared to low oil content ecotype tissues (Shiraz-17 & Shiraz-21) (Supplementary Table S4). Similarly, unigenes with a higher expression in low oil content ecotype (Shiraz-17 & Shiraz-21) were gathered in Clusters 5 and 15 (Supplementary Table S5). Unigenes presented in Clusters 9 and 10 displayed approximately equal expression patterns in genotypes Arak-10, Shiraz-17 and Shiraz-21. The heatmaps of classified differentially expressed unigenes which had differential expression patterns in inflorescence tissues of the two ecotypes (Clusters 5, 8, 11 and 15) are shown in Supplementary Fig. S3.

### GO classification of differentially expressed genes

**Figure 6:**
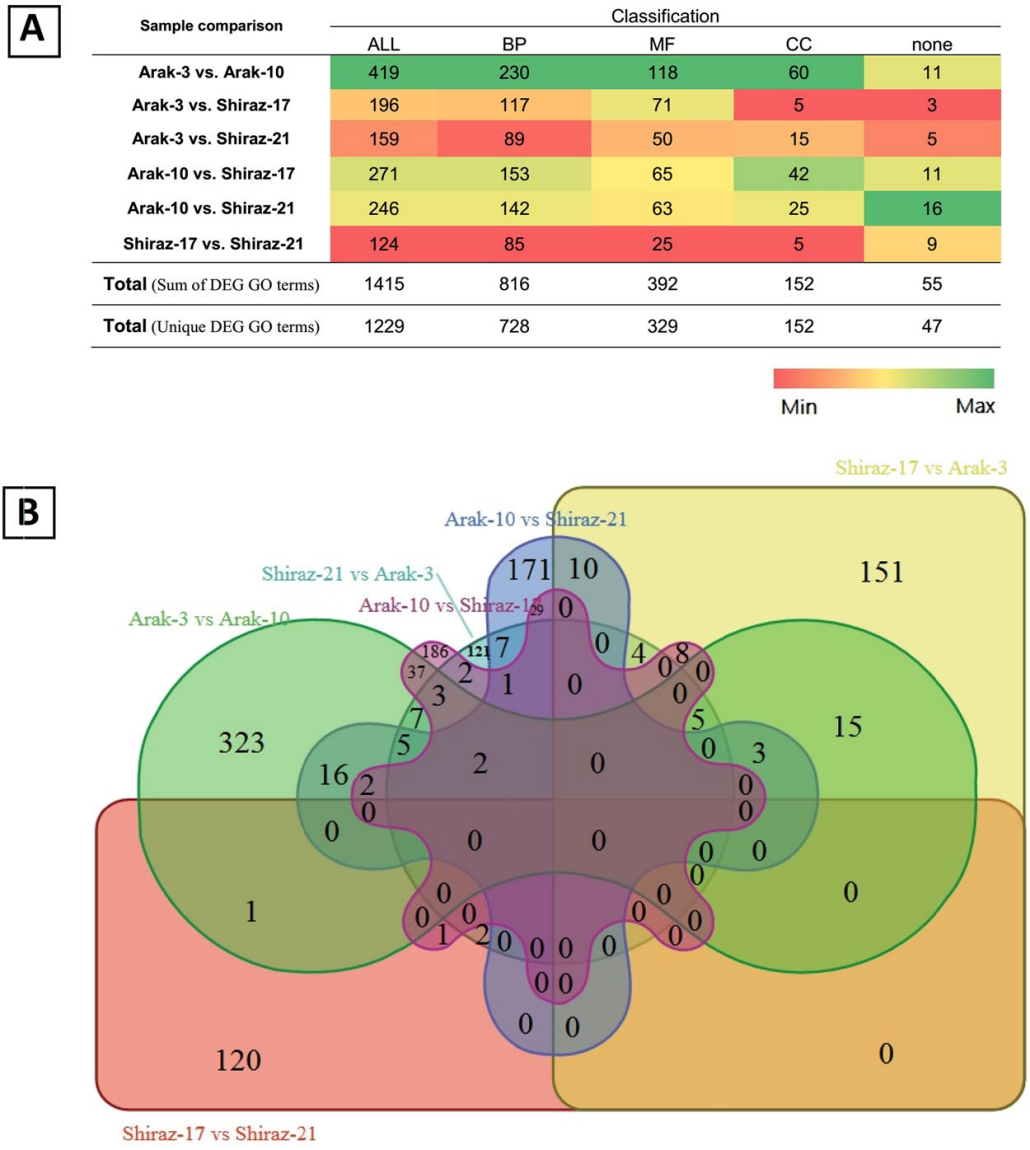
GO annotation categories of differentially expressed genes between 4 genotypes. (**A**) Total number of differentially expressed GO terms between each pair genotypes (All) and Classification of the differentially expressed GO terms between each pair genotypes in three main categories: cellular component (CC), molecular function (MF) and biological process (BP). (**B**) Venn diagram of 6 set data of differentially expressed GO terms between each pair genotypes.

Gene ontology classification showed that the differentially expressed unigenes were classified into 55 functional categories (Supplementary Fig. S4). The dominant (≥50%) categories were ‘cell’ and ‘cell part’, in the cellular component class of ontology and ‘binding’ in the molecular function class of ontology, and also ‘metabolic process’ and ‘cellular process’ in the biological process class of ontology. There were 65 (4.22%) differentially expressed unigenes related to the secondary metabolic process, categorized into 31 GO terms (Supplementary Table S6). The total number of GO terms related to the differentially expressed genes in all comparisons was 1415, which 1229 GO terms were unique (Fig. 6A). A comparison of the differentially expressed GO terms of four genotypes of the ajowan inflorescence of two ecotypes showed that Shiraz-17 and Shiraz-21 had only 124 differentially expressed GO terms, indicating that these two genotypes had more similar gene expression patterns (Fig. 6A). A classification of the GO terms into three main categories indicated that, in all comparison of pair genotypes, the largest differentially expressed GO term category was the biological process (BP), followed by molecular function (MF) and cellular component (CC), respectively (Fig. 6A). The differentially expressed GO terms from four genotypes produced six sets of data, which are shown in a Venn diagram (Fig. 6B). Among all the sets, three GO terms were related to the secondary metabolite, GO:0019748, as a secondary metabolic process in Arak-3 vs. Arak-10 and Arak-3 vs. Shiraz-17 sets, GO:0090487 as a secondary metabolite catabolic process in set Arak-3 vs. Arak-10, and GO:0044550 as a secondary metabolite biosynthetic process in set Arak-3 vs. Shiraz-17 (Supplementary Dataset). The number of unigenes in the GO:0019748 category was 531, of which 17 and 8 differentially expressed genes were in this category in Arak-3 vs. Arak-10 and Arak-3 vs. Shiraz-17 sets, respectively (Supplementary Dataset). There were 10 GO terms related to the terpenoids process, represented only in four sets: Arak-10 vs. Shiraz-17, Arak-10 vs. Shiraz-21, Arak-3 vs. Shiraz-17 and Arak-3 vs. Shiraz-21 (Supplementary Table S7). These showed that the two ajowan ecotypes (Arak and Shiraz) had different expression and regulation of the genes related to the terpenoids process.

### GO enrichment of differentially expressed genes

**Figure 7:**
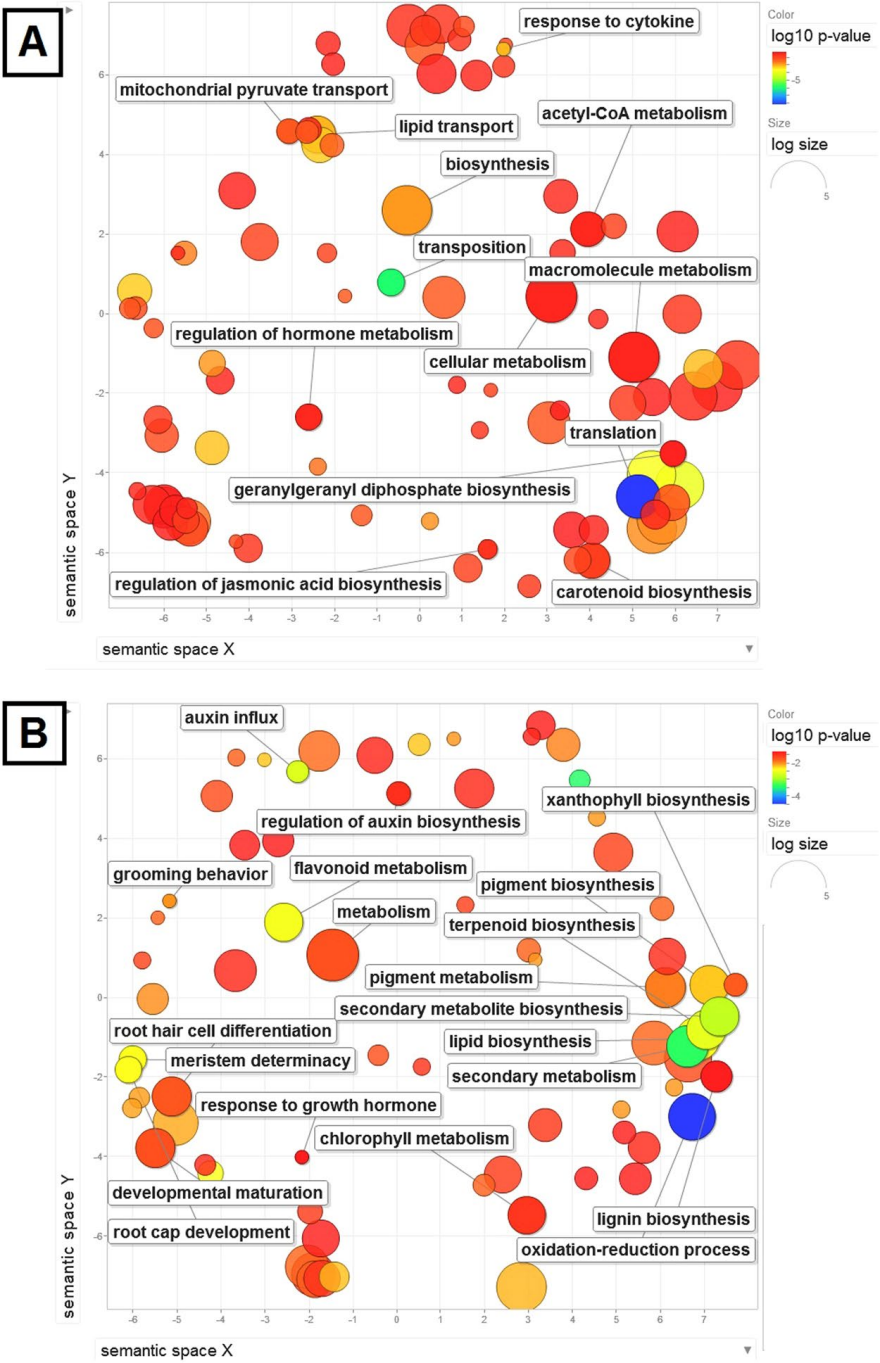
GO category enrichment analysis of differentially expressed unigenes related to biological process found in (**A**) Arak-3 vs. Shiraz-17 and (**B**) Arak-10 vs. Shiraz-17 combinations. Circles depicted by filled color show significantly enriched GO terms with log10 p-value < 0.05. The colour and the size of bubbles show the p-value (legend in upper right-hand corner) and the frequency of the GO term in the underlying GOA database in REVIGO analysis, respectively (bubbles of more general terms are larger).

GO enrichment analysis was done on the differentially expressed unigenes of each pair set to obtain over-represented GO categories of unigenes. In all, 1415 GO terms were used for enrichment from four genotypes (Fig. 6A). In the biological process class, the enriched categories were related to the metabolic processes of macromolecule metabolism, secondary metabolism, carotenoid biosynthesis, root development, fruit ripening, and response to growth hormones (Fig. 7 and Supplementary Fig. S5). The presence of GO categories related to terpenoid biosynthesis (Fig. 7A), geranylgeranyl diphosphate biosynthesis, and acetyl-CoA metabolism (Fig. 7B) led us to identify the genes related to differential terpenoid biosynthesis in two ecotypes.

**Figure 8:**
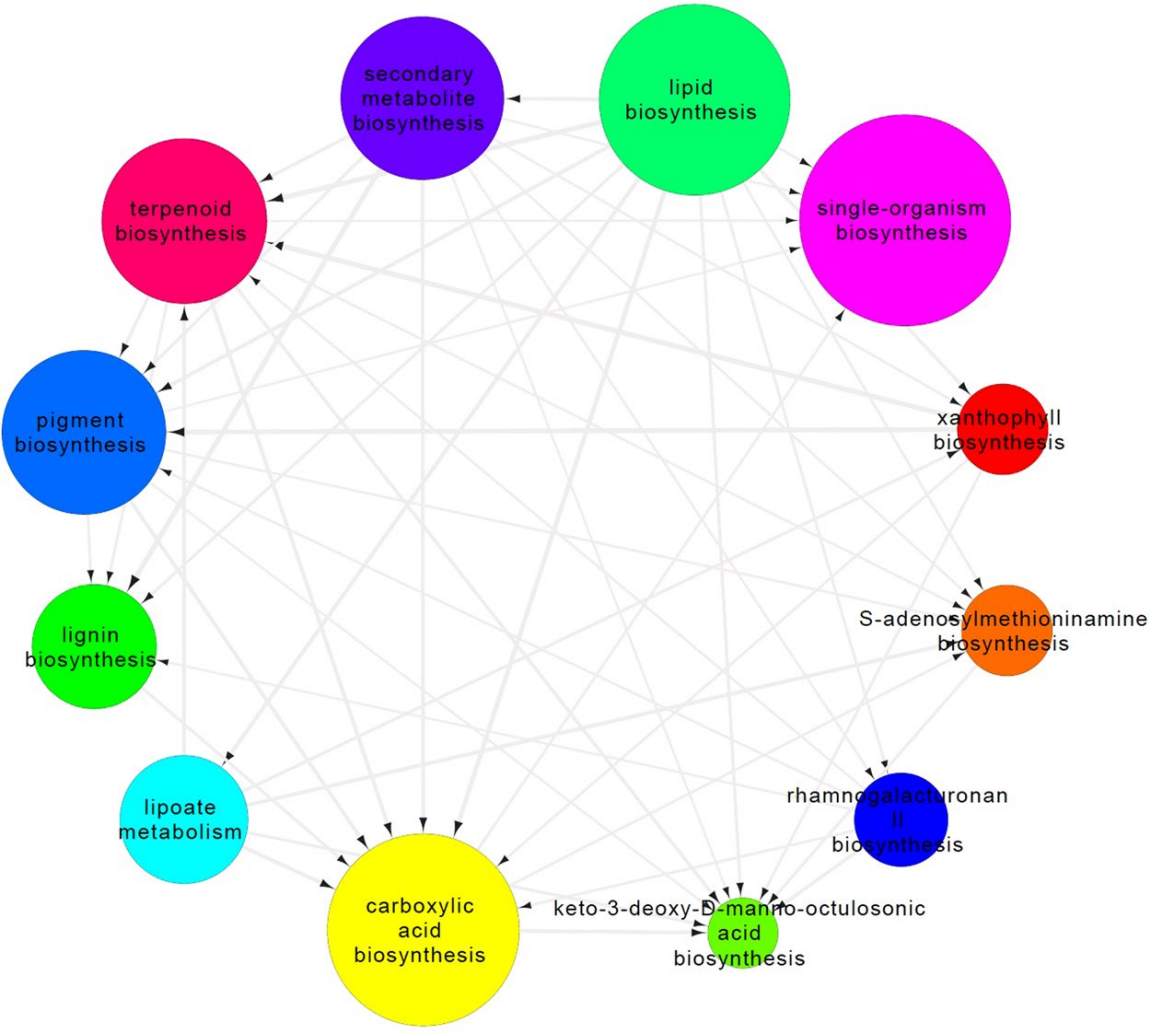
Selected first neighbours of terpenoid biosynthesis node of the Interactive graph view of GO category enrichment analysis of differentially expressed genes related to the biological process found in inflorescence tissues of Arak-3 vs. Shiraz-17 genotypes produced by REVIGO and adjusted by Cytoscape. Bubble color indicate the GO term name; bubble size indicates the log size of the GO term.

Enriched categories in the molecular function class were oxidoreductase, geranyl transtransferase, and cytomchrome_c oxidase activity, which are probably related to putative terpenoid-encoding genes (Supplementary Fig. S6). In the cellular component class, enriched categories of cytoplasmic, cytosolic plastid, chloroplast, and thylakoid parts were observed (Supplementary Fig. S7). The interactive graph view of enriched GO terms was constructed for Arak-3 vs. Shiraz-17 (Fig. 8 and Supplementary Fig. S8), and Arak-10 vs. Shiraz-17 (Supplementary Fig. S9).

### Identification of unigenes involved in biosynthesis of monoterpenoids

**Figure 9:**
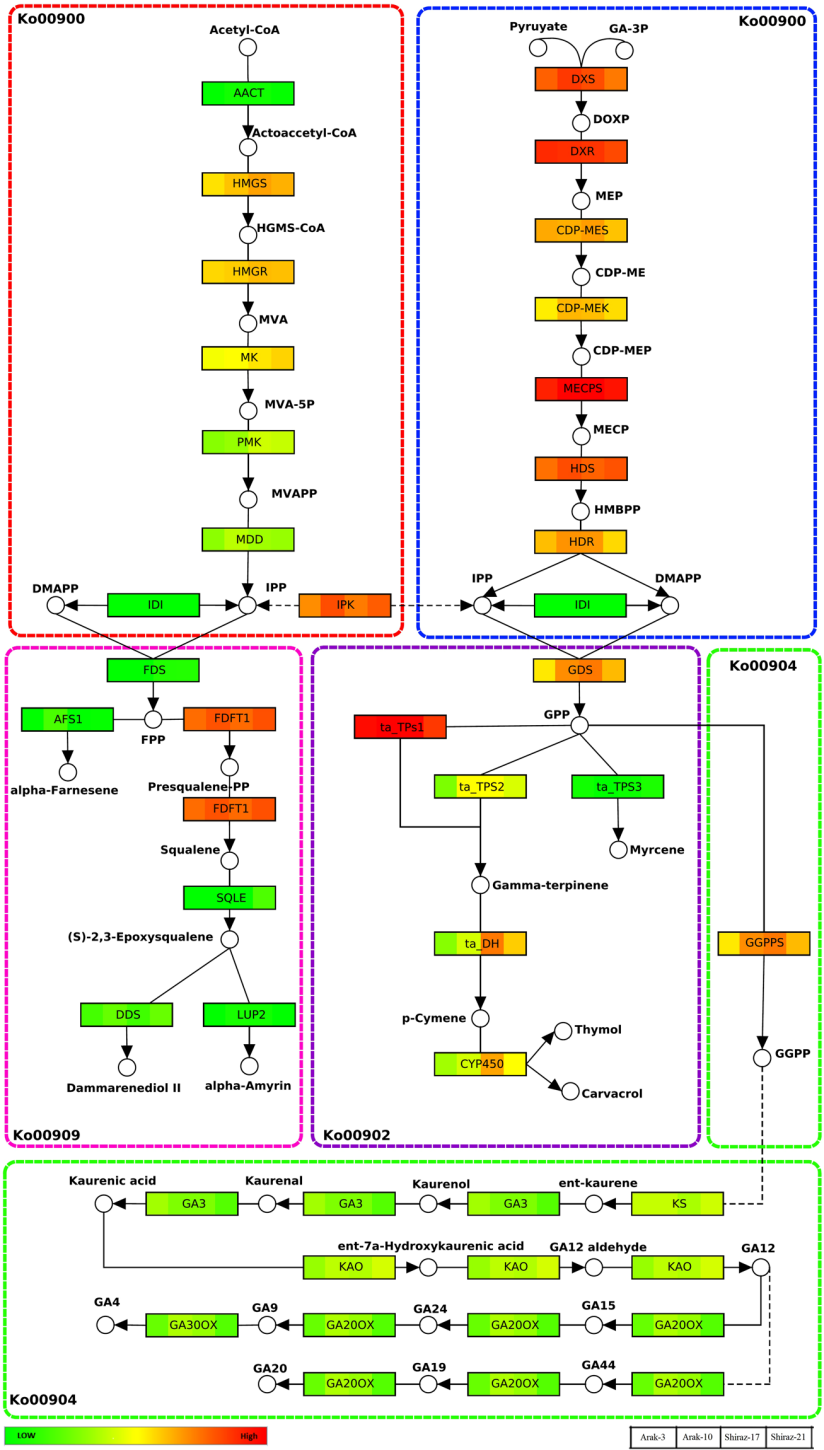
Expression patterns of *T. ammi* unigenes involved in the terpenoid biosynthetic pathways. The ko00902 section is an assumption by the authors according to our data and literature reviews. Sum of FPKMs of all transcripts related to each gene is used. Solid arrows represent established biosynthetic steps, whereas broken arrows illustrate the involvement of multiple enzymatic reactions. Ko numbers show the KEGG maps code related to each pathway. DMAPP, dimethylallyl pyrophosphate; DXS, 1-deoxy-D-xylulose 5-phosphate synthase; DXR, 1-deoxy-D-xylulose 5-phosphate reductoisomerase; FPP, farnesyl pyrophosphate; FPS, FPP synthase; GA, gibberellin; GA20ox, GA 20-oxidase; GA30ox, GA 30-oxidase; GGPP, geranylgeranyl pyrophosphate; GGPS, GGPP synthase; GPP, geranyl pyrophosphate; GDS, GPP synthase; HMG-CoA, hydroxymethylglutaryl-CoA; HMGR, HMG-CoA reductase; HMGS, HMG-CoA synthase; IPI, isopentenyl pyrophosphate isomerase; IPP, isopentenyl pyrophosphate; KAO, ent-kaurenoic acid oxidase; TPS, terpene synthase; MCT, 2-C-methyl-D-erythritol 4-phosphate cytidylyltransferase; MK, mevalonate kinase; MDD, mevalonate diphosphate decarboxylase; PMK, phosphomevalonate kinase.

Biosynthesis of monoterpenoids in ajwoan utilizes the terpenoid backbone pathway as an intermediate. This pathway consists of two steps; the final product of the first step is IPP, produced from MVA and MEP pathways. The products of the second step are GPP and FPP, depending on MEP or MVA pathway, produced from IPP (Fig. 9). In this study, unigenes related to all the enzymes of the terpenoid backbone pathway, were identified in the inflorescence transcriptome of ajowan. The data showed that each enzyme was encoded by multiple copies of unigenes (Supplementary Table S8), differentially expressed in four genotypes of ajowan (Fig. 9). In the terpenoid backbone biosynthesis pathway (ko00900), the maximum number of unigenes was assigned to AACT (11 unigenes), followed by HDR (9 unigenes), and HMGR (8 unigenes), whereas for HMGS, IPK, IDI, CDP-MES, CDP-MEK, MECPS, and FDS, only one unigene was observed (Supplementary Table S8). Among the identified TPS genes in the monoterpenoid biosynthesis pathway (ko00902), the maximum number of unigenes was identified for *ta*_TPS2 (8 unigenes) followed by *ta*_TPS1 (6 unigenes) and *ta*_TPS3 (6 unigenes), respectively (Supplementary Table S8). In the diterpenoid biosynthesis pathway (ko00904), the maximum number of unigenes was identified for GA2OX (18 unigenes) followed by GA20OX (9 unigenes), GA3 (6 unigenes) and CPS-KS (6 unigenes), respectively (Supplementary Table S8). In the sesquiterpenoid and triterpenoid biosynthesis pathway (ko00909), the maximum number of unigenes was identified in case of PSM (8 unigenes) followed by SQLE (7 unigenes) (Supplementary Table S8).

TPS unigenes obtained from the Trinity assembly having complete CDS were utilized to analyze the expression pattern of each unigene. The differential expression patterns obtained from four different genotypes were validated by the QRT-PCR analysis of the selected TPS unigenes using both ecotypes of ajowan (Table 4). Among the four selected TPS unigenes, the 56475 unigene (*ta*_TPS2) had the maximum relative expression in the inflorescence tissue of ajowan (Table 4). The transcript expression of 56475 (*ta*_TPS2) and 37637 (*ta*_TPS1) unigenes were significantly up-regulated in the Arak ecotype compared to the Shiraz ecotype (Table 4).

**Table 4:** Results of relative expression of four TPS genes in *T. ammi* produced by REST 2009, V2.0.13.

Unigene	Gene	Type	Reaction Efficiency	Expression	Std. Error	95% C.I.	P(H1)	Result
37637	TPS1	TRG	0.825	3.958	3.516–4.456	3.483–4.497	0.000	UP
40869	TPS2	TRG	0.8125	0.119	0.089–0.162	0.079–0.179	0.000	DOWN
56475	TPS2	TRG	0.89	9.201	7.473–11.504	6.724–12.644	0.000	UP
19758	TPS1	TRG	0.9375	0.323	0.222–0.487	0.187–0.562	0.000	DOWN
36245	SAND	REF	0.8475	0.989				
47069	eIF-4a	REF	0.775	1.011				

Legend: P(H1) - Probability of alternate hypothesis that difference between sample and control groups is due only to chance. TRG - Target REF – Reference.

### Identification of gene families involved in biosynthesis of monoterpenoids

Cyclic monoterpenes such as thymol are the final products of different secondary transformations including isomerization-cyclization and hydroxylation of GPP as a substrate^38,39^. The gene families involved in the biosynthesis of monoterpenoids (Fig. 9) might be terpene synthases (TPS), cytochrome P450s (CYP450s)^40^, dehydrogenase (DHs)^17,41^, and transcription factors (TFs)^42,43^, which may provide the biosynthesis of thymol in ajowan. The differentially expressed members of these gene families in ajowan transcriptome were identified by using annotated unigenes, some of which were putatively involved in the monoterpenoids biosynthesis pathway (Supplementary Table S9). The results of annotation using TAIR10 and carrot genome databases showed that 203 unigenes were of the CYP450 gene family, while 25 unigenes were differentially expressed in inflorescence tissues of four genotypes (Supplementary Table S9). Some of these might be involved in the biosynthesis of thymol and other monoterpenoids. Altogether 38 unigenes were annotated as Terpene synthases (TPs), of which four unigenes were differentially expressed in inflorescence tissues of four genotypes (Supplementary Table S9, Suplementary_Dataset_2). It was found that 1230 unigenes were related to the dehydrogenase (DHs) gene family and 53 unigenes among them showed differential expression in four genotypes (Supplementary Table S9). The blastx against *Panax quinquefolius* showed 38 identified terpene synthase (TPS) unigenes in *T. ammi* (Supplementary Table S9) had high similarity with 18 sequence IDs in *P. quinquefolius*, which 8 sequence IDs had functional annotation in *P. quinquefolius* (Supplementary Table S14).

### Analysis of transcription factor genes related to terpenoid biosynthesis

**Figure 10:**
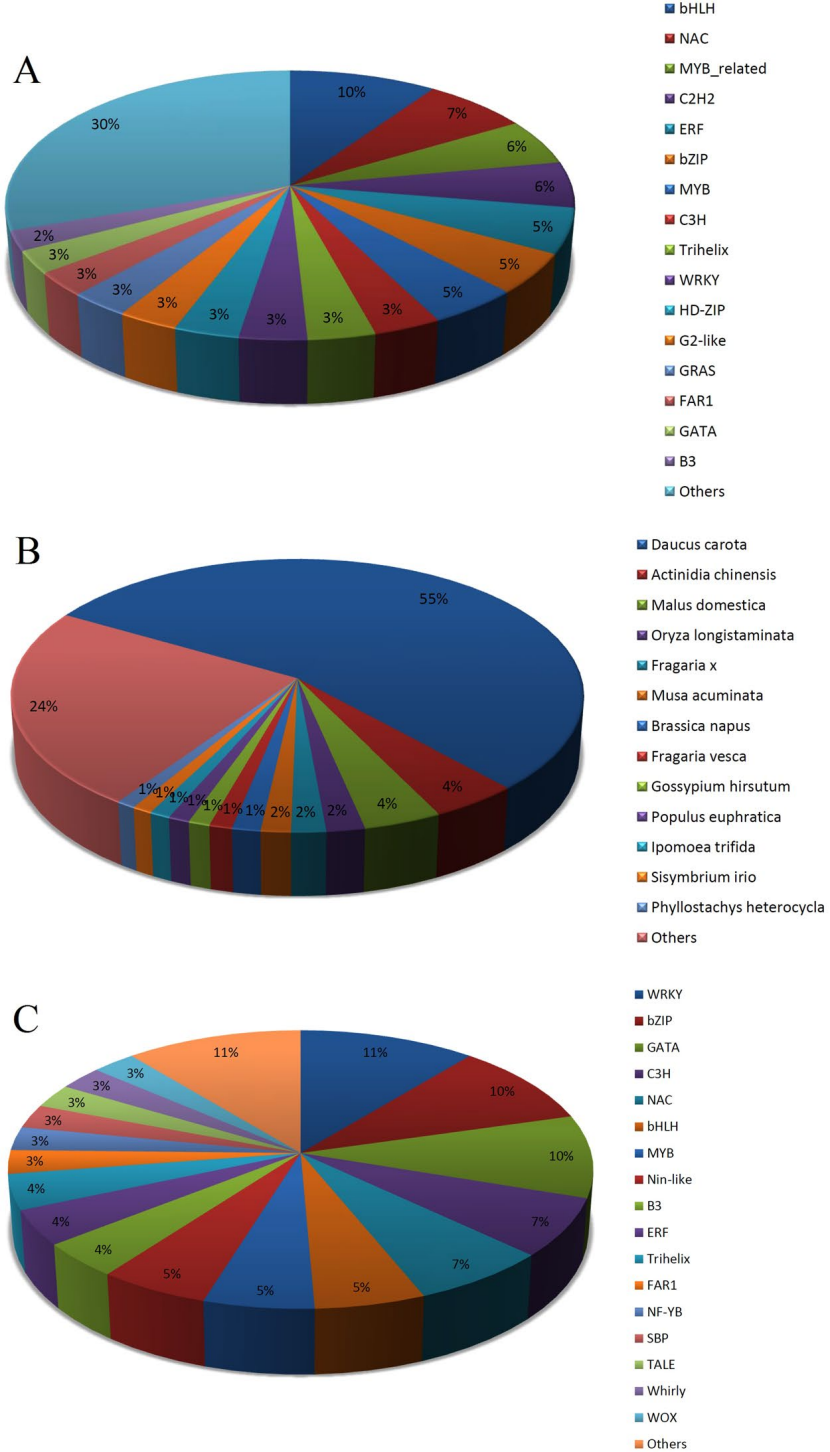
Transcription factor genes of *T. ammi*. (**A**) Percent of identified TFs families. (**B**) Percent of TFs genes had high homology with plant species. (**C**) Percent of differentially expressed TFs families.

**Figure 11:**
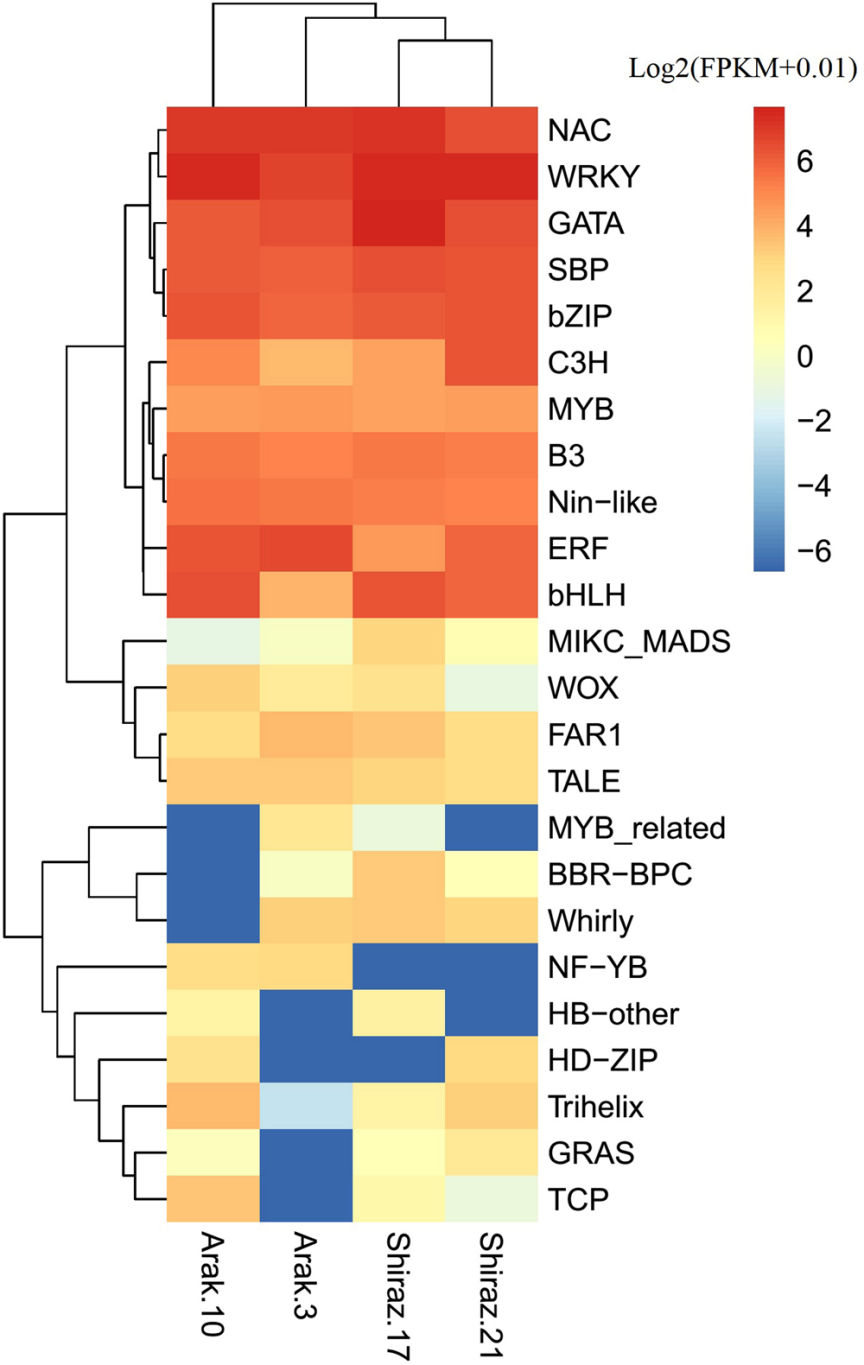
Heatmap of differentially expressed TFs families.

The BLAST x search against Plant TF database identified 1831 unigenes (Supplementary Table S9) (2586 unitranscripts, Supplementary Table S10) as a putative transcription factor (TFs) distributed in 56 families having high homology (identity N80%) with 182 plant species TFs (Supplementary Table S11). In our study, the dominant families were bHLH, NAC, MYB-related, C2H2, ERF, bZIP, MYB, C3H, Trihelix, and WRKY (Fig. 10A). More than half (55%) of *T. ammi*’s TFs had high homology with *Daucus carota* TFs (Fig. 10B). Seventy-three unigenes, belonging to 24 TFs families expressed differentially in the inflorescence tissues (Supplementary Table S9), included WRKY, bZIP, GATA, C3H, NAC, bHLH, and MYB families (Fig. 10C). The expression pattern of all differentially expressed TF gene families in inflorescence tissues of *T. ammi* is represented in Fig. 11. Among putative transcription factors (1831 unigenes) identified in *T. ammi* there were 1343 unigenes (73%) in results of BLASTx against Medicinal Plant Genomics Resource (MPGR) protein database (Supplementary Fig. S10). Between 14 medicinal plants of this database, *Panax quinquefolius* (605, 45%) had the most number of similar TF to *T. ammi* (Supplementary Fig. S10). Also from 1831 identified transcription factors (TFs) unigenes in *T. ammi* (Supplementary Table S9), 1797 TFs had similarity to 1344 sequence IDs in *P. quinquefolius*, which among them 432 sequence IDs had functional annotation in *P. quinquefolius*. This high similarity could be due to the close relatedness in taxonomy of *P. quinquefolius* and *T. ammi* also *P. quinquefolius* was diverged form Apiaceae family approximately 66 million years ago (Xu, *et al*. 2017). The high similarity of TF genes of *T. ammi* with *Panax quinquefolius* may be due to the near genetic relationship of this two medicinal plant species.

### Pathway enrichment analysis for differentially expressed genes

A pathway enrichment analysis of differentially expressed genes helped us to identify significant KEGG metabolic pathways, which included significantly more expressed genes. There were 10 KEGG pathways identified as significantly enriched, with a cutoff p-value 10e-3, in at least one of the pairwise genotype comparison (Table 5). There was only one enriched pathway in the pairwise genotypes comparison of Shiraz-17 vs. Shiraz-21. The most common enriched pathways in terms of all pairwise genotype comparisons were ‘Metabolites biosynthesis’ and ‘Biosynthesis of secondary metabolites’. ‘Glycerophospholipid metabolism’ and ‘Glyoxylate and dicarboxylate metabolism’ enriched pathways were only observed in pairwise genotype comparisons between two ecotypes.

**Table 5:** KEGG pathway enrichment analysis of differentially expressed genes pairwise genotypes comparison.

Pairwise genotypes comparison	Enriched Pathway Term	ID	Input number	Background number	P-Value^*,**^
Arak-3 vs. Arak-10	Metabolic pathways	ath01100	248	1910	7.9E-11
	Protein processing in endoplasmic reticulum	ath04141	41	212	5.0E-06
	Biosynthesis of secondary metabolites	ath01110	131	1076	4.5E-05
	Endocytosis	ath04144	27	142	2.4E-04
	Oxidative phosphorylation	ath00190	28	162	7.2E-04
Arak-10 vs. Shiraz-17	Metabolic pathways	ath01100	150	1910	8.1E-06
	Protein processing in endoplasmic reticulum	ath04141	27	212	1.1E-04
	Glycerophospholipid metabolism	ath00564	15	86	2.0E-04
Arak-10 vs. Shiraz-21	Protein processing in endoplasmic reticulum	ath04141	25	212	3.6E-04
	Metabolic pathways	ath01100	136	1910	3.7E-04
Shiraz-17 vs. Arak-3	Metabolic pathways	ath01100	228	1910	8.8E-11
	Biosynthesis of secondary metabolites	ath01110	129	1076	1.1E-06
	Flavonoid biosynthesis	ath00941	9	21	1.5E-04
	Glyoxylate and dicarboxylate metabolism	ath00630	16	74	5.0E-04
	Carbon metabolism	ath01200	36	262	1.1E-03
	Glycerophospholipid metabolism	ath00564	16	86	1.1E-03
Shiraz-17 vs. Shiraz-21	Arginine biosynthesis	ath00220	8	35	1.4E-04
Shiraz-21 vs. Arak-3	Metabolic pathways	ath01100	226	1910	6.2E-09
	Biosynthesis of secondary metabolites	ath01110	122	1076	1.2E-04
	Glycerophospholipid metabolism	ath00564	18	86	5.1E-04
	Glyoxylate and dicarboxylate metabolism	ath00630	16	74	7.5E-04

^*^Statistical test method: hypergeometric test/Fisher’s exact test.

^**^FDR correction method: Benjamini and Hochberg.

## Discussion

The inflorescence of ajowan (*Trachyspermum ammi*) is a rich repository of secondary metabolites, especially monoterpens, such as thymol. Phytochemical analyses of inflorescence tissues of Arak and Shiraz ecotypes showed that three components—thymol, γ-terpinene, and p-cymene—to be the main components of ajowan essential oils, comprising 98% of essential oil components in the studied ecotypes. In other reports, too, the major components of the essential oils of ajowan were thymol, γ-terpinene and p-cymene^21,44^. Secondary metabolite biosynthesis and accumulation were tissue-specific and related to enzymes and regulator genes, demonstrating tissue-specific expression patterns^22,45^. Hence, the inflorescence tissues from two Iranian native ecotypes (Arak and Shiraz) were selected for transcriptome analysis. Phytochemical analyses of the inflorescence tissues (Fig. 1C) showed differences in the amount of the main components of the essential oils. These quantitative variations in the essential oil content can be due to the terpene synthase and other secondary metabolite related gene expression and regulation. The valuable data generated by the RNA-Seq analysis of the *T. ammi* transcriptome can be used to explain the thymol biosynthesis pathway, paralogues of genes and gene families related to thymol biosynthesis. The result of annotation indicated that more than 48% of the assembled unigenes of ajowan matched with the genomic databases of other plants. The unigenes, identified in this research, had a higher annotation percentage against the carrot genome database compared to other plant databases (Fig. 3A). This may be due to this fact that both carrot and ajowan are members of the Apiaceae family. The results of this research indicated the high similarity of assembled unigenes of *T. ammi* from Apiaceae family with *Panax quinquefolius* (American ginseng) from Araliaceae family^46^ (Fig. 3B), which illustrated this fact that both two families are from the same order. Both Araliaceae and Apiaceae families are from Apiales order and they may be the remnants of an ancient group of pro-araliads^47^.

The diversity of the GO terms related to the assembled unigenes, as demonstrated by functional GO assignments, showed the variety of the unigenes involved in the secondary metabolite biosynthesis pathway (Fig. 4). Furthermore, the detection of novel genes related to secondary metabolite processes may be possible from *T. ammi* RNA-seq data. A large number of unigenes involved in secondary metabolite processes in *T. ammi* was identified by the mapping of unigenes against the KEGG pathway database (Tables 2 and 3). A total of 2316 unigenes, including isoprenoid and putative terpenoid pathway genes, were involved in the secondary metabolite biosynthesis. The differentially expressed unigenes (2,626) of four different genotypes of *T. ammi* were represented by a differential gene expression analysis (Fig. 5). In all the fifteen clusters, unigenes grouped in clusters 5, 8, 11 and 15, as shown in Fig. 5, were expressed in different pattern in the high oil content ecotype (Arak-3 & Arak-10) compared to low oil content ecotype (Shiraz-17 & Shiraz-21) and might be involved in secondary metabolite biosynthesis. The presence of HDS gene (29437_0_2) and two transcription factors (56455_2_2 and 41958_0_3) in cluster 8, might be one of the causes of the increase essential oil amount in Arak ecotype (Supplementary Table S15). From 1531 DEGs unigenes classified by GO database (Supplementary Table S2), 65 differentially expressed unigenes (4.22%) were related to secondary metabolic process and categorized into 31 GO terms (Supplementary Table S6), suggesting that the differences in essential oil contents between four ajowan genotypes could have resulted from these genes. A comparison of the differentially expressed GO terms showed that genotype Shiraz-17 and Shiraz-21 had more similar gene expression patterns and regulation among four genotypes (Fig. 6). The differentially expressed GO terms, which related to the terpenoids process, are represented in four sets—Arak-10 vs. Shiraz-17, Arak-10 vs. Shiraz-21, Shiraz-17 vs. Arak-3 and Shiraz-21 vs. Arak-3. It can be concluded on the basis of the results that Arak and Shiraz ajowan ecotypes have different expressions and regulation patterns for genes related to the terpenoids process, probably due to different genetic backgrounds of the two ecotypes.

The summarization and clustering of GO terms present enriched GO clusters related to secondary metabolism processes, terpenoid biosynthesis, geranylgeranyl diphosphate biosynthesis, and acetyl-CoA metabolism particularly, leading us to the identification of genes related to differential terpenoid biosynthesis in the two ecotypes (Fig. 7, Supplementary Figs S5, S6 and S7). The interactive graph of the GO category’s enrichment analysis (Fig. 8) showed that terpenoid biosynthesis and secondary metabolite biosynthesis GO terms were directly linked to lipid biosynthesis GO term, highlighting the importance of those GO terms in secondary metabolite biosynthesis. Terpenoids are lipids and their production is biochemically dependent on sugar, amino acid, and triacylglycerol synthesis and catabolism through isoprene unit^48^. The terpenoid biosynthetic pathway, in other plants, has been studied and a lot of participating genes have been detected in this pathway^49–51^. Thymol biosynthesis utilizes the intermediates of terpenoid backbone^52^.

The KEGG pathway enrichment analysis identified 112 unigenes involved in the terpenoid backbone biosynthesis pathway (Table 3). For the terpenoid backbone pathway, more than one unigene for each enzymatic step was detected from annotation of unigenes. Hence, these unigenes could be part of one larger gene or members of the multigene families. This analysis may allow the detection of most of the paralogue genes, encoding the catalyzing enzymes for different steps. The number of identified unigenes for AACT, HDR and HMGR genes were 11, 9 and 8, respectively (Supplementary Table S8), suggesting these unigenes might have undergone various gene duplication events. These unigenes have been identified in other plant species producing terpenoid compounds such as *Arabidopsis*^53^, *Gentiana macrophylla*^54^, *Phyllanthus amarus*^37^, *Gossypium raimondii* and *Glycine max*^55^. The second step of the monoterpene biosynthetic pathway from GDS toward specific thymol biosynthesis in *T. ammi* is still uncharacterized. Accordingly, an effort has been made to detect the main genes and gene families involved in this step of thymol biosynthesis (Supplementary Tables S8, S9 and S13). The annotation results showed that three TPS genes were involved in monoterpenoid biosynthesis in ajowan (Fig. 9). QRT-PCR results showed two unigenes (56475 (*ta*_TPS2) and 37637 (*ta*_TPS1)) with complete CDS were significantly up-regulated in Arak ecotype compared to Shiraz ecotype, which might be the responsible unigenes for quantitative variation of monoterpene between two ecotypes. Based on the annotation results, 203 unigenes were identified as CYP450 gene family members. Similarly, the Arabidopsis genome contains 272 genes belonging to the CYP450 family. In our study, identification of differentially expressed unigenes related to CYP450s (25 unigenes) represented involvement of some of CYP450s genes in the thymol biosynthesis. Some of the CYP450s participate in essential oil biosynthesis^56,57^. In the menthol pathway in mint, a CYP450 responsible for the hydroxylation of the monoterpene limonene has been described^56^. Considering that thymol is an aromatic and hydroxylated compound, it is conceivable that cytochrome P450 enzymes are responsible for its formation. However, a definite proof of certain P450 enzymes that are able to catalyze the complete reaction still remains unknown. The formation of thymol in Thyme and Oregano species was also in the focus of a study^16^. C. Crocoll isolated the sequences of five cytochrome P450 enzymes from *Origanum vulgare* and *Thymus vulgaris*. It was shown that the expression of these genes correlated with the occurrence of thymol and carvacrol in the essential oils of thyme and oregano. Therefore a role in monoterpene biosynthesis can be hypothesized^16^. The relevance of cytochrome P450 genes to thymol biosynthesis was also shown in another study^57^. In this study, two P450 enzymes were investigated in order to clarify their role in the formation of thymol and carvacrol from γ-terpinene. In spite of some differences in details, all the mentioned studies demonstrate the role of CYP enzymes in hydroxylation of γ-terpinene and final production of thymol.

In this study, differentially expressed unigenes related to TFs gene families in inflorescence tissues of *T. ammi* were identified (Fig. 10C). Unigenes of NAC, WRKY, GATA, SBP, bZIP, bHLH, ERF, C3H, MYB, B3, and Nin-like TFs gene families had a high level of expression in inflorescence tissues of *T. ammi* (Fig. 11). In plants, secondary metabolism pathways have been found to be regulated by several transcription factor families including NAC, WRKY, ERF, MYB, and bHLH^58,59^. Identification of TFs regulating secondary metabolism pathways would be a powerful generic tool for plant metabolic engineering in ajowan. In general, this study gives rise to a valuable genomic resource data to explore tissue-specific thymol biosynthesis based on terpenoid diversity in future.

## Methods

### Plant Material

Ajowan ecotypes ‘Arak’ and ‘Shiraz’ of *Trachyspermum ammi* (L.), respectively, containing different amount of oil content and thymol yield among 23 indigenous ecotypes gathered from various parts of Iran^21^, were grown at the experimental station of College of Abouraihan, University of Tehran, Pakdasht, Tehran, Iran. Seeds of two ecotypes were obtained from the gene bank of the Research Institute of Forests and Rangelands (RIFR) and planted in transplanting trays in the greenhouse in March 2014 and transplanted to the experimental field. Samples were collected from the inflorescence tissue (~5 days after anthesis^60^) of four genotypes (2 plants in each ecotype) and immediately frozen in liquid nitrogen and stored at −80 °C (Fig. 2A,B).

### Phytochemical analysis

The analysis of ajowan oils from inflorescence tissue of four genotypes was carried out using GC/MS. The GC/MS analysis was performed on a GC/MS apparatus using HP (Agilent Technology): 6890 Network GC System gas chromatograph connected to a mass detector (5973 Network Mass Selective Detector). The gas chromatograph was equipped with an HP-5MS capillary column (fused silica column, 30 m × 0.25 mm i.d., Agilent Technologies) and an EI mode with ionization energy of 70 eV with a scan time of 0.4 s and mass range of 40–460 amu was used. 1 μl of diluted samples (1/100; v/v, in methanol) was manually injected in the splitless mode. The interface temperature was 290 °C. Helium gas was selected as the carrier with the same flow rate as GC/FID. The program of the oven temperature was initiated at 40 °C, held for 1 min. then raised up to 250 °C at the rate of 3 °C/min. The oil compounds were identified by a comparison of their retention indices (RI), mass spectra fragmentation with NIST (National Institute of Standards and Technology) Adams libraries spectra, and Wiley 7 n.1 mass computer library, and with those reported in literature^61^.

### RNA-Seq library construction and sequencing

The entire RNA was extracted from *T. ammi* inflorescences using the TRIzol reagent (Invitrogen, USA) according to the manufacturer’s instruction. The RNA samples were treated with DNase I (TURBO DNase; Ambion, TX, USA). The quality and quantity of the extracted RNA were assessed with 1% agarose gel and NanoDrop 1000 spectrophotometer (Thermo Scientific, USA), respectively. Furthermore, subsequent quality control for the extracted RNA was examined by using a QC Bioanalyzer (Agilent Technologies, Hørsholm, Denmark) and the RNA integrity number (RIN) of each sample was greater than 8. The selection of Poly A, cDNA preparation, adapter ligation, formation of clusters and sequencing was performed at the Beijing Genomes Institute (China), according to the manufacturer’s recommendation, with the use of standard Illumina kits. The sequencing was done on an Illumina HiSeq. 2000 platform with a paired-end and read length of 101 nt.

### RNA-Seq data processing and de novo assembly

Raw reads were subjected to quality control using the Trimmomatic software (Version 0.36)^62^ to filter out adaptor and low-quality nucleotide/sequences. After trimming, FastQC (http://www.bioinformatics.babraham.ac.uk/projects/fastqc/) was used to examine the characteristics of the libraries and to verify the trimming efficiency. The high-quality filtered reads were used for downstream analyses. De novo assembly of the resulting pooled clean reads was conducted using Trinity (Release 2016-03-17)^63^ and default parameters and kmer length of 25 (Fig. 2C). Resulted Trinity transcripts were clustered using CAP3 package by identity cutoff 95% and min overlap of 250 nt. Clustered assemblies and singletons were called unigenes, which represented putatively identified genes and the resultant sequences of Trinity were called unitranscripts, representing putative transcript isoforms. To identify the candidate Coding Sequence (CDS) regions within all transcript sequences, we used the TransDecoder tool (http://transdecoder.github.io) which is an ORF predictor. Protein sequences of predicted ORFs were used for annotation and functional analysis using databases such as KEGG and Uniprot. The identified unigenes from annotation result were checked for completeness of CDS.

### Functional annotation of *T. ammi* assembled unigenes

The functional annotation of the ajowan transcriptome was done using a Trinotate annotation pipeline (http://trinotate.github.io/). Furthermore, the assembled unigenes of ajowan were blasted against non-redundant (NR) proteins (http://www.ncbi.nlm.nih.gov/), UniProt (Swiss-Prot and TrEMBL)^64^, Arabidopsis (version TAIR10)^65^, Carrot protein (https://www.ncbi.nlm.nih.gov/genome/?term=txid4039[orgn])^66^ and Medicinal Plant Genomics Resource (MPGR) protein (http://medicinalplantgenomics.msu.edu/species_list.shtml) databases with E-value cutoff of 10e-5. Metabolic pathways and functional descriptions for each ajowan unigene were assigned using the Kyoto Encyclopedia of Genes and Genome (KEGG, http://www.genome.jp/kegg/)^67^ by KEGG Automatic Annotation Server (KAAS, http://www.genome.jp/kegg/kaas/)^68^. GO functional classification for all assembled unigenes was represented by the WEGO software^69^.

### Differential gene expression analysis

Abundance of Trinity assembled transcripts were estimated by the RSEM^70^ software (Fig. 2C). First, the original high-quality reads were aligned back to the assembled transcriptome using Bowtie (version 1.1.2)^63^, then RSEM was run to estimate the number of reads aligned to each transcript. Normalized expression values for each unitranscript and each unigene were included in the RESM output files. Differentially expressed (DE) contigs were identified from the counts matrix estimated by RSEM through the Bioconductor package edgeR^71^ using Rstudio^72^. The edgeR package using TMM normalization to adjust for any differences in sample composition. To obtain the differentially expressed genes (DEGs), a threshold false discovery rate (FDR) of ≤ 0.001 and an absolute value of log2Ratio ≥ 2 and four-fold change were used. For DEGs clustering, first, expression values (FPKM) were log2 transformed and median-centered by unigenes. Then Hierarchical clustering of DE transcripts was obtained. Unigenes clusters, extracted from the hierarchical clustering using R. for partitioning unigenes into clusters the Ptree method (cut tree based on this percent of max(height) of tree) was used. The GO category enrichment analysis for DEGs was performed using goseq.^73^ and REVIGO^74^ (http://revigo.irb.hr/) and its interactive graph view adjusted by the Cytoscape^75^ software (version 3.4.0). The pathway enrichment analysis for DEGs was carried out using KOBAS 2.0^76^. Significant pathways were identified by using Fisher’s exact test and corrected P values < 0.001.

### Identification of unigenes and gene families related to terpenoid biosynthesis

Unigenes involved in the triterpenoid backbone biosynthesis and monoterpenoid biosynthesis pathways were identified based on the annotation results. Metabolic pathway construction and visualization was performed by Pathvisio3 software^77^. CYP450, TPs, DHs and the TF gene family members were retrieved from the assembly and annotated results using in-house scripts. All identified unigenes and gene family members related to terpenoid biosynthesis were assessed and curated manually, using BLAST against NCBI and Uniprot databases. Differentially expressed unigenes of ajowan ecotypes was shown as a heatmap using the pheatmap package in RStudio^72^ software. Calculation and visualization of Venn diagrams were performed by online software (http://bioinformatics.psb.ugent.be/webtools/Venn/).

### Quantitative real-time PCR analysis

Quantitative expression of selected unigenes according RNA-Seq analysis of inflorescences tissues of ajowan ecotypes were carried out using the Real Time PCR Detection System (Corbett Rotor-Gene 6000 instrument, Corbett Life Science, Australia) and TaKaRa SYBR^®^ Green Permix Ex Taq^TM^ II. Two internal control genes (SAND and eIF-4a) from *T. ammi* were used for estimating the relative transcript level of the analyzed unigenes. The REST software^78^ (http://rest-2009.gene-quantification.info/) was used for data analysis of qRT-PCR amplification. Two technical replicates were used for all the qR-TPCR experiments. Specific oligonucleotides of selected genes for qRT-PCR analysis are shown in Supplementary Table S12.

## Electronic supplementary material

Supplementary file

Supplementary Datafile_1

Supplementary Datafile_2

## Data Availability

All data generated during this study are included in this published article and its supplementary information files. Furthermore, the raw sequencing data of genotypes have been deposited in the NBCI (https://www.ncbi.nlm.nih.gov) under bioproject codes: PRJNA359623 and PRJNA362991; biosample accession numbers SRR5137050, SRR5137051, SRR5137053 and SRR5137052.
