# Supplementary material for: Comparative transcriptome analysis to identify putative genes involved in thymol biosynthesis pathway in medicinal plant Trachyspermum ammi L
Source: Sci Rep. 2018 Sep 7;8:13405. doi: 10.1038/s41598-018-31618-9 (PMC6128898; doi:10.1038/s41598-018-31618-9)
Supplement: Supplementary file 1 — Supplementary file [file 41598_2018_31618_MOESM1_ESM.doc]

Scientific Reports Supporting Information

**Comparative transcriptome analysis to identify putative genes involved in thymol biosynthesis pathway in medicinal plant *Trachyspermum ammi* L*.***

Mehdi Soltani Howyzeh^1^, Seyed Ahmad Sadat Noori^1^, Vahid Shariati J.^2,3^ and Mahboubeh Amiripour^1^

1- Department of Agronomy and Plant Breeding Science, College of Abouraihan, University of Tehran, Tehran, Iran.

2- Molecular Biotechnology Department, National Institute of Genetic Engineering and Biotechnology, Tehran, Iran.

3-NIGEB Genome Center, National Institute of Genetic Engineering and Biotechnology, Tehran, Iran.

**Corresponding Authors:**

**1-** Vahid Shariati J., Ph.D.

Plant Molecular Biotechnology Department, National Institute of Genetic Engineering and Biotechnology, Tehran, Iran

NIGEB Genome Center, National Institute of Genetic Engineering and Biotechnology, Tehran, Iran.

**E-mail:** [vshariati@nigeb.ac.ir](mailto:vshariati@nigeb.ac.ir)

**2-** Seyed Ahmad Sadat Noori, Ph.D.

Department of Agronomy and Plant Breeding Science, College of Abouraihan, University of Tehran, Tehran, Iran

**E-mails:** [noori@ut.ac.ir](mailto:noori@ut.ac.ir)


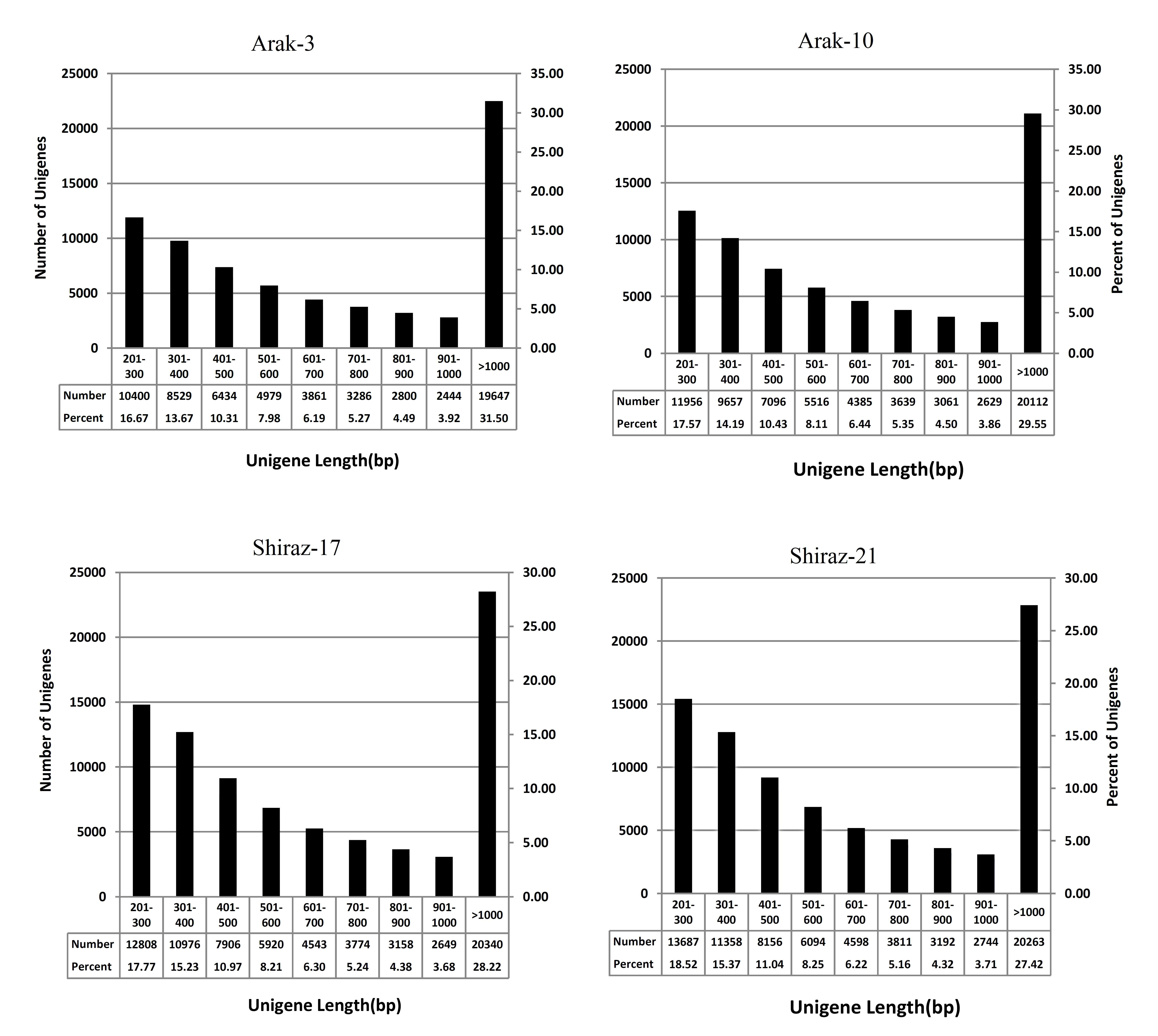


**Supplementary Figure S1**: Length distribution of four genotypes of the ajowan unigenes *de novo* assembled by Trinity.


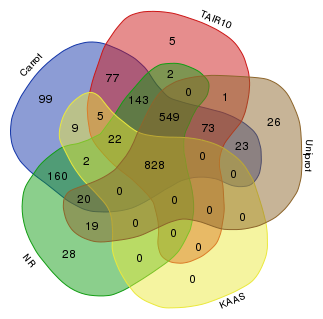


**Supplementary Figure S2:** Venn diagram of BLAST annotation results of differentially expressed unigenes against different databases.


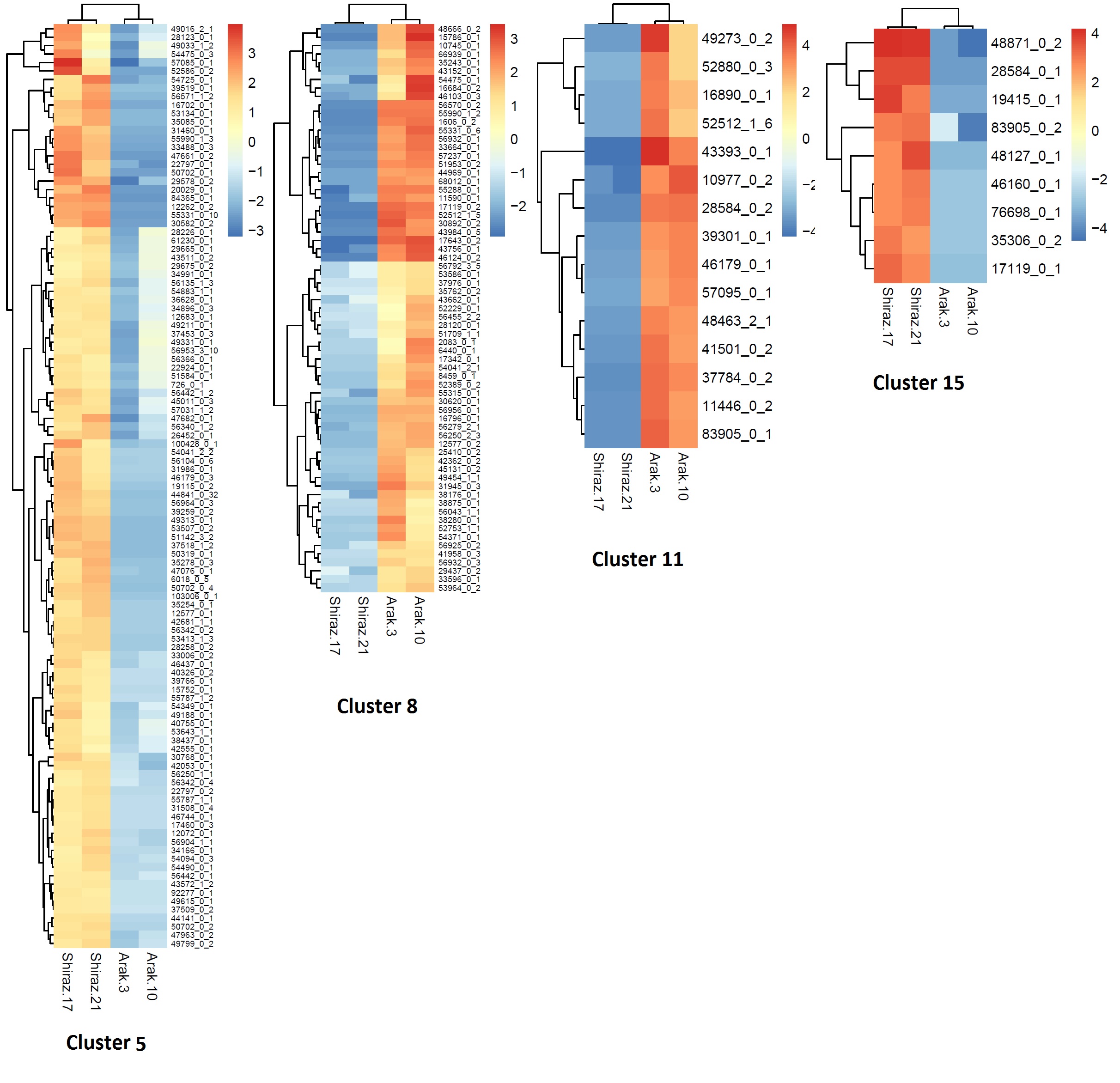


**Supplementary Figure S3:** The heatmaps of clusters 5, 8, 11 and 15 of the classified differentially expressed unigenes which had differential expression patterns in inflorescence tissues of two ajowan ecotypes


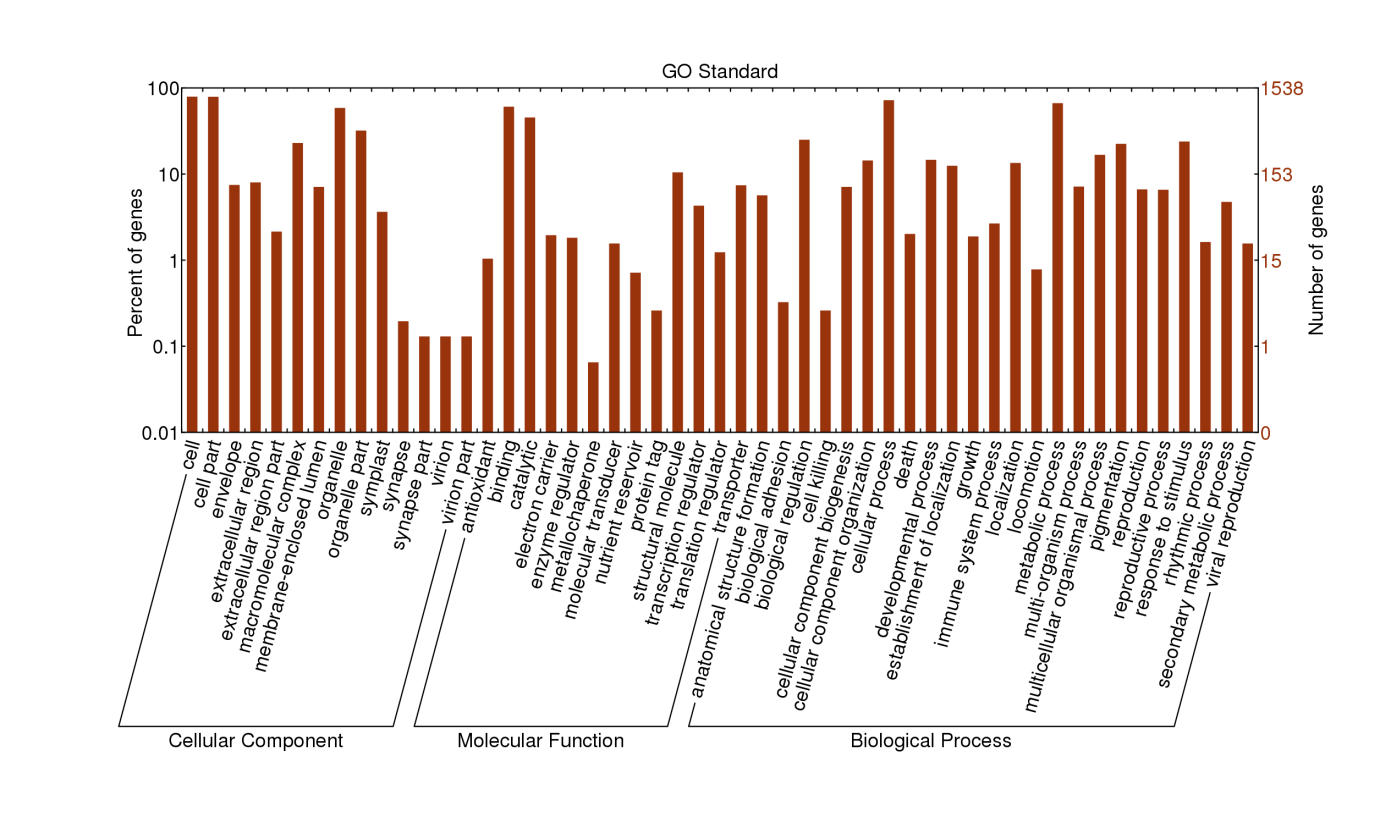


**Supplementary Figure S**4: GO functional classification of the differentially expressed unigenes. Bars represent the percent and number of assignments of unigenes to each GO term. Plot with Y axis in log (10) scale.


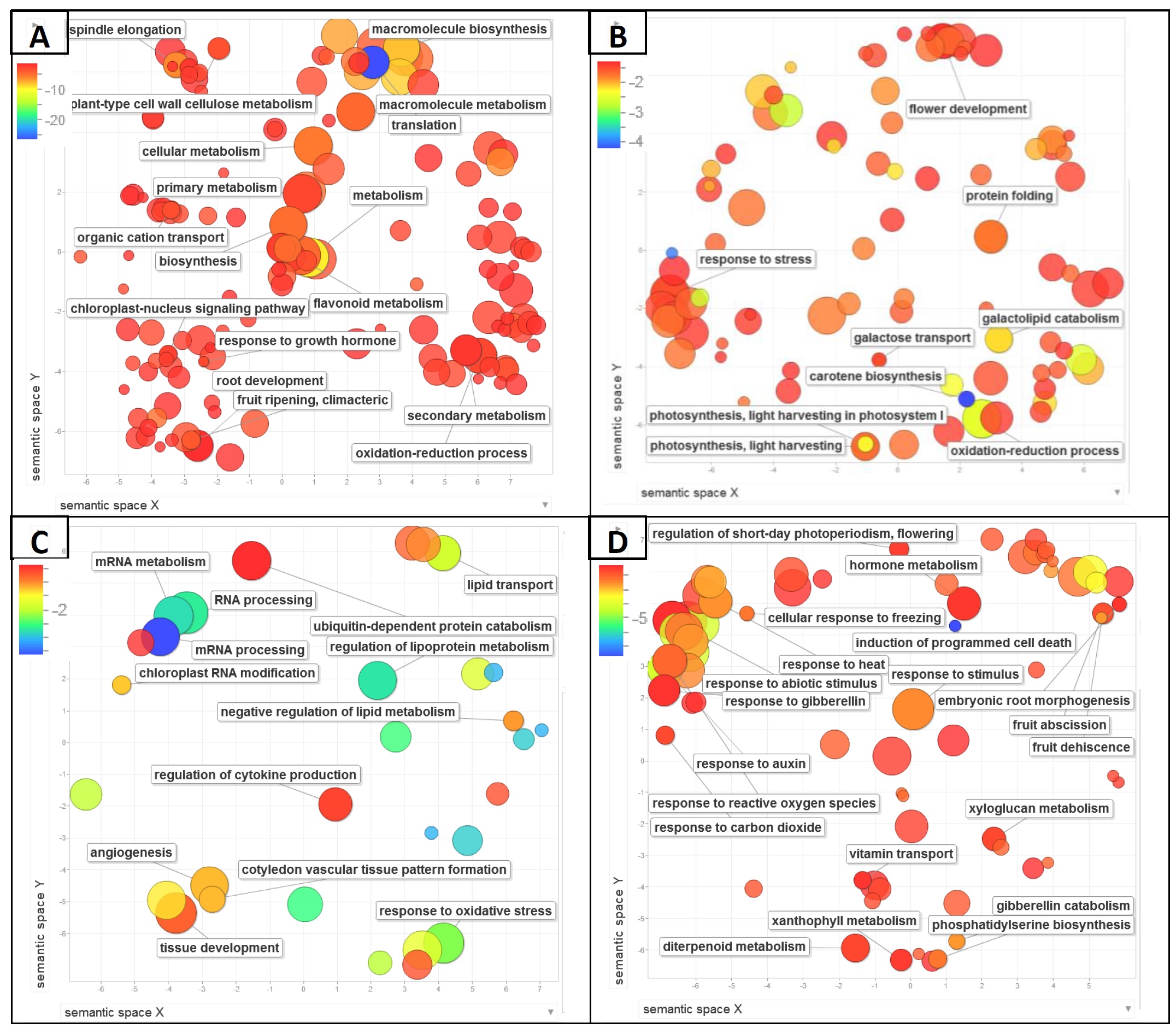


**Supplementary Figure S**5: **GO category enrichment analysis of differentially expressed unigenes related to the biological process found in A) Arak-3 vs. Arak-10, B) Arak-10 vs. Shiraz-21, C) Shiraz-17 vs. Shiraz-21 and D) Arak-3 vs. Shiraz-21 combinations**. Circles depicted by filled color show significantly enriched GO terms with log10 p-value <0.05. The colour and the size of bubbles show the p-value (legend in upper left-hand corner) and the frequency of the GO term in the underlying GOA database in REVIGO analysis, respectively (bubbles of more general terms are larger).

**
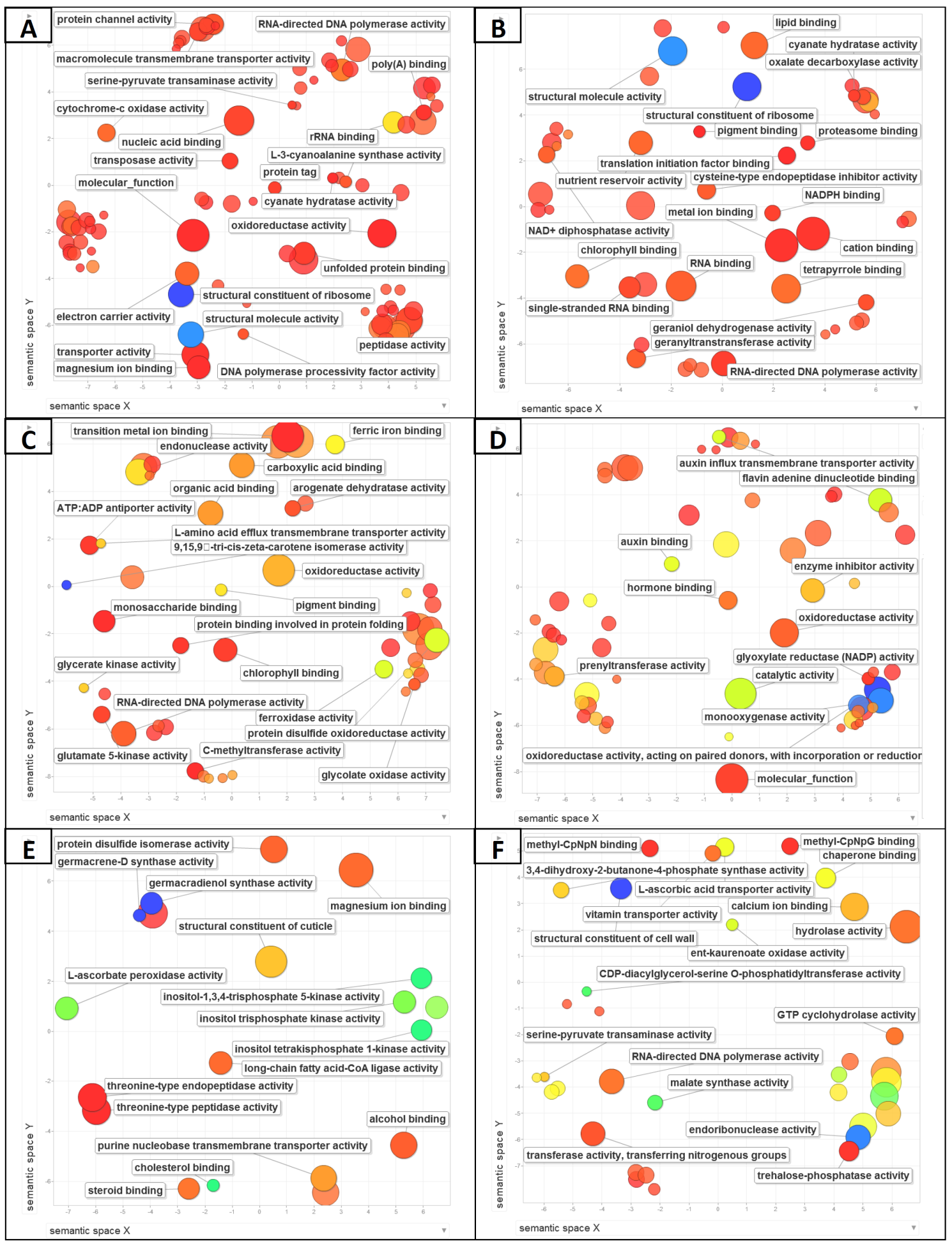
** **Supplementary Figure S6: GO category enrichment analysis of differentially expressed genes related to the molecular function found in 6 combinations of four inflorescence tissues.** Circles depicted by filled color show significantly enriched GO terms with log10 p-value <0.05. The colour and the size of bubbles show the p-value (legend in upper left-hand corner) and the frequency of the GO term in the underlying GOA database in REVIGO analysis, respectively (bubbles of more general terms are larger). **A**) Arak-3 vs. Arak-10. **B**) Arak-10 vs. Shiraz-17. **C**) Arak-10 vs. Shiraz-21. **D**) Arak-3 vs. Shiraz-17. **E**) Shiraz-17 vs. Shiraz-21. **F**) Arak-3 vs. Shiraz-21.


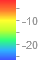

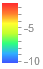

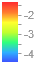

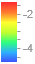

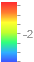

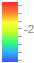


**
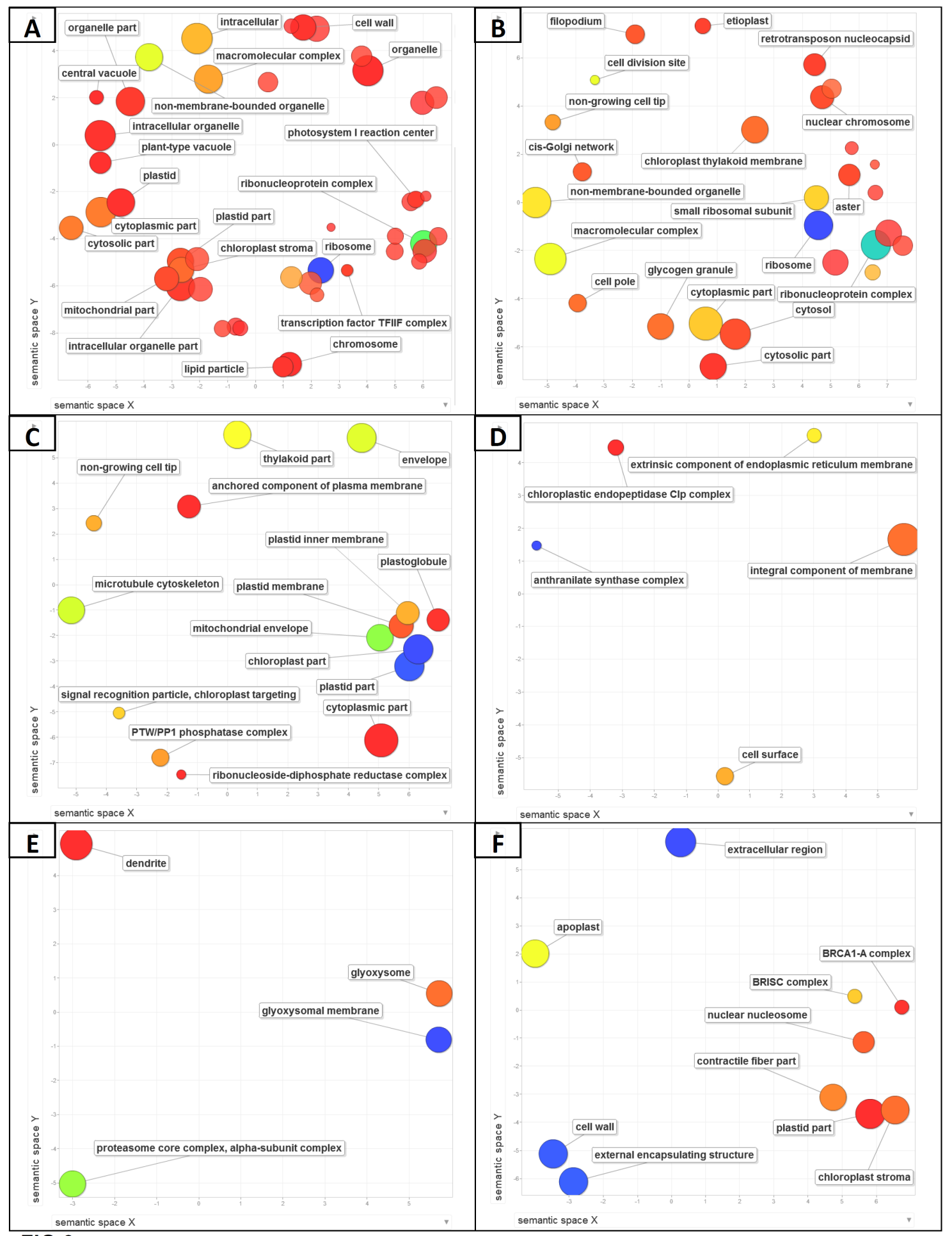
**


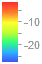

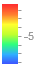

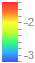

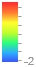

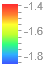

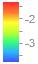


**Supplementary Figure S7: GO category enrichment analysis of differentially expressed genes related to the cellular component found in 6 combinations of four inflorescence tissues.** Circles depicted by filled color show significantly enriched GO terms with log10 p-value <0.05. The colour and the size of bubbles show the p-value (legend in upper left-hand corner) and the frequency of the GO term in the underlying GOA database in REVIGO analysis, respectively (bubbles of more general terms are larger). **A**) Arak-3 vs. Arak-10. **B**) Arak-10 vs. Shiraz-17. **C**) Arak-10 vs. Shiraz-21. **D**) Arak-3 vs. Shiraz-17. **E**) Shiraz-17 vs. Shiraz-21. **F**) Arak-3 vs. Shiraz-21.

**Supplementary Figure S8:** Interactive graph view of GO category enrichment analysis of the differentially expressed genes related to the biological process found in inflorescence tissues of Arak-3 vs. Shiraz-17 genotypes produced by REVIGO and adjusted by Cytoscape. The colour of bubbles shows the p-value of the GO term in the underlying GOA database, respectively. Highly similar GO terms are linked by edges in the graph, where the line width indicates the degree of similarity.

A

B

**Supplementary Figure S9: A) Interactive graph view of GO category enrichment analysis of differentially expressed genes related to the biological process found in inflorescence tissues of Arak-10 vs. Shiraz-17 genotypes produced by REVIGO and adjusted by Cytoscape.** The colour of bubbles shows the p-value of the GO term in the underlying GOA database, respectively. Highly similar GO terms are linked by edges in the graph, where the line width indicates the degree of similarity. **B) Selected first neighbours of geranylgeranyl diphosphate biosynthesis node of Fig. S9A.** Bubble color indicate the GO term name; bubble size indicates the log size of the GO term.

**Supplementary Figure S10:** Percent of identified TFs genes had high homology with medicinal plant sequences of Medicinal Plant Genomics Resource (MPGR) database.

**Supplementary Table S1:** Overview of assembled unigenes for each library

| Features | libraries | | | |
| --- | --- | --- | --- | --- |
|  | Arak-3 | Arak-10 | Shiraz-17 | Shiraz-21 |
| Number of Unigenes | 62380 | 68051 | 72074 | 73903 |
| Max. length (bp) | 9627 | 7872 | 9460 | 8863 |
| Average Length (bp) | 892 | 859 | 840 | 826 |
| Q1 of Length (bp) | 357 | 349 | 344 | 339 |
| Q2 of Length (bp) | 621 | 596.37 | 571 | 558 |
| Q3 of Length (bp) | 1205 | 1140 | 1103 | 1079 |
| Sum of Length (bp) | 55693146 | 58483783 | 60560849 | 61063698 |
| Max. effective_length (bp) | 9580 | 7820 | 9405 | 8810 |
| Max. expected_count | 1914722 | 2081981 | 1568454 | 2533048 |
| Average expected_count | 161 | 146 | 170 | 150 |
| Sum of expected_count | 10043507 | 9984795 | 12261294 | 11093126 |
| Max. TPM | 44301 | 46604 | 27114 | 53135 |
| Average TPM | 16.03 | 14.69 | 13.87 | 13.53 |
| Max. FPKM | 30108.83 | 32958.84 | 20239.55 | 36127.15 |
| Average FPKM | 10.90 | 10.39 | 10.36 | 9.20 |

**Supplementary Table S2**: Annotation summary of assembled unigenes and differentially expressed unigenes against different databases

| Unigenes | Databases | | | | | | Total number of unique unigenes |
| --- | --- | --- | --- | --- | --- | --- | --- |
|  | KAAS | Uniprot | NR | TAIR10 | Carrot | GO |  |
| All assembled unigenes | 20,018 | 40,137 | 48,899 | 45,188 | 56,264 | 39,877 | 59,910 |
| Differentially expressed unigenes | 866 | 1,539 | 1,773 | 1,705 | 2,010 | 1,538 | 2,091 |

**Supplementary Table S3:** Unigenes from the assembly were mapped to the reference canonical pathways in KEGG using KAAS

| NO. | Reference Pathway (ko) | N. of Unigenes | NO. | Reference Pathway (ko) | N. of Unigenes | NO. | Reference Pathway (ko) | N. of Unigenes | NO. | Reference Pathway (ko) | N. of Unigenes | NO. | Reference Pathway (ko) | N. of Unigenes |
| --- | --- | --- | --- | --- | --- | --- | --- | --- | --- | --- | --- | --- | --- | --- |
| 1 | ko00010 | 332 | 72 | ko00591 | 53 | 143 | ko02010 | 92 | 214 | ko04341 | 83 | 285 | ko04961 | 94 |
| 2 | ko00020 | 159 | 73 | ko00592 | 146 | 144 | ko02020 | 61 | 215 | ko04350 | 86 | 286 | ko04962 | 69 |
| 3 | ko00030 | 142 | 74 | ko00600 | 90 | 145 | ko03008 | 238 | 216 | ko04360 | 97 | 287 | ko04964 | 21 |
| 4 | ko00040 | 264 | 75 | ko00603 | 19 | 146 | ko03010 | 1033 | 217 | ko04370 | 57 | 288 | ko04966 | 55 |
| 5 | ko00051 | 168 | 76 | ko00604 | 25 | 147 | ko03013 | 448 | 218 | ko04380 | 64 | 289 | ko04970 | 33 |
| 6 | ko00052 | 134 | 77 | ko00620 | 238 | 148 | ko03015 | 325 | 219 | ko04390 | 131 | 290 | ko04971 | 31 |
| 7 | ko00053 | 110 | 78 | ko00623 | 3 | 149 | ko03018 | 290 | 220 | ko04391 | 105 | 291 | ko04972 | 78 |
| 8 | ko00061 | 131 | 79 | ko00624 | 50 | 150 | ko03020 | 90 | 221 | ko04392 | 33 | 292 | ko04973 | 19 |
| 9 | ko00062 | 84 | 80 | ko00625 | 38 | 151 | ko03022 | 77 | 222 | ko04510 | 92 | 293 | ko04974 | 15 |
| 10 | ko00071 | 166 | 81 | ko00626 | 15 | 152 | ko03030 | 154 | 223 | ko04520 | 66 | 294 | ko04975 | 15 |
| 11 | ko00072 | 14 | 82 | ko00627 | 58 | 153 | ko03040 | 563 | 224 | ko04530 | 66 | 295 | ko04976 | 79 |
| 12 | ko00073 | 58 | 83 | ko00630 | 195 | 154 | ko03050 | 184 | 225 | ko04540 | 91 | 296 | ko04977 | 17 |
| 13 | ko00100 | 55 | 84 | ko00640 | 102 | 155 | ko03060 | 118 | 226 | ko04550 | 34 | 297 | ko04978 | 30 |
| 14 | ko00130 | 68 | 85 | ko00643 | 12 | 156 | ko03070 | 27 | 227 | ko04611 | 40 | 298 | ko05010 | 319 |
| 15 | ko00140 | 91 | 86 | ko00650 | 52 | 157 | ko03320 | 143 | 228 | ko04612 | 197 | 299 | ko05012 | 307 |
| 16 | ko00190 | 395 | 87 | ko00660 | 17 | 158 | ko03410 | 90 | 229 | ko04614 | 12 | 300 | ko05014 | 83 |
| 17 | ko00195 | 123 | 88 | ko00670 | 57 | 159 | ko03420 | 157 | 230 | ko04620 | 94 | 301 | ko05016 | 414 |
| 18 | ko00196 | 51 | 89 | ko00680 | 153 | 160 | ko03430 | 115 | 231 | ko04621 | 69 | 302 | ko05020 | 64 |
| 19 | ko00220 | 77 | 90 | ko00710 | 211 | 161 | ko03440 | 129 | 232 | ko04622 | 21 | 303 | ko05030 | 1 |
| 20 | ko00230 | 365 | 91 | ko00720 | 98 | 162 | ko03450 | 16 | 233 | ko04623 | 46 | 304 | ko05031 | 76 |
| 21 | ko00232 | 17 | 92 | ko00730 | 22 | 163 | ko03460 | 126 | 234 | ko04626 | 433 | 305 | ko05032 | 16 |
| 22 | ko00240 | 276 | 93 | ko00740 | 18 | 164 | ko04010 | 153 | 235 | ko04630 | 33 | 306 | ko05033 | 14 |
| 23 | ko00250 | 132 | 94 | ko00750 | 20 | 165 | ko04011 | 147 | 236 | ko04650 | 56 | 307 | ko05034 | 208 |
| 24 | ko00253 | 18 | 95 | ko00760 | 51 | 166 | ko04012 | 50 | 237 | ko04660 | 49 | 308 | ko05100 | 84 |
| 25 | ko00254 | 5 | 96 | ko00770 | 62 | 167 | ko04013 | 144 | 238 | ko04662 | 68 | 309 | ko05110 | 119 |
| 26 | ko00260 | 147 | 97 | ko00780 | 33 | 168 | ko04014 | 127 | 239 | ko04664 | 42 | 310 | ko05120 | 84 |
| 27 | ko00261 | 20 | 98 | ko00785 | 11 | 169 | ko04015 | 79 | 240 | ko04666 | 172 | 311 | ko05130 | 103 |
| 28 | ko00270 | 261 | 99 | ko00790 | 31 | 170 | ko04020 | 124 | 241 | ko04668 | 24 | 312 | ko05131 | 72 |
| 29 | ko00280 | 122 | 100 | ko00791 | 1 | 171 | ko04022 | 163 | 242 | ko04670 | 22 | 313 | ko05132 | 74 |
| 30 | ko00281 | 1 | 101 | ko00830 | 61 | 172 | ko04024 | 178 | 243 | ko04710 | 85 | 314 | ko05133 | 124 |
| 31 | ko00290 | 40 | 102 | ko00860 | 62 | 173 | ko04062 | 55 | 244 | ko04711 | 25 | 315 | ko05134 | 219 |
| 32 | ko00300 | 17 | 103 | ko00900 | 112 | 174 | ko04064 | 90 | 245 | ko04712 | 112 | 316 | ko05140 | 80 |
| 33 | ko00310 | 88 | 104 | ko00901 | 5 | 175 | ko04066 | 149 | 246 | ko04713 | 48 | 317 | ko05142 | 105 |
| 34 | ko00330 | 132 | 105 | ko00902 | 41 | 176 | ko04068 | 195 | 247 | ko04720 | 104 | 318 | ko05143 | 15 |
| 35 | ko00340 | 42 | 106 | ko00903 | 73 | 177 | ko04070 | 155 | 248 | ko04721 | 168 | 319 | ko05145 | 214 |
| 36 | ko00350 | 109 | 107 | ko00904 | 57 | 178 | ko04071 | 179 | 249 | ko04722 | 196 | 320 | ko05146 | 35 |
| 37 | ko00360 | 86 | 108 | ko00905 | 35 | 179 | ko04072 | 150 | 250 | ko04723 | 52 | 321 | ko05152 | 242 |
| 38 | ko00361 | 3 | 109 | ko00906 | 76 | 180 | ko04075 | 502 | 251 | ko04724 | 96 | 322 | ko05160 | 78 |
| 39 | ko00362 | 15 | 110 | ko00908 | 82 | 181 | ko04080 | 1 | 252 | ko04725 | 22 | 323 | ko05161 | 101 |
| 40 | ko00363 | 50 | 111 | ko00909 | 44 | 182 | ko04110 | 325 | 253 | ko04726 | 20 | 324 | ko05162 | 203 |
| 41 | ko00364 | 3 | 112 | ko00910 | 75 | 183 | ko04111 | 223 | 254 | ko04727 | 79 | 325 | ko05164 | 206 |
| 42 | ko00380 | 123 | 113 | ko00920 | 51 | 184 | ko04112 | 32 | 255 | ko04728 | 139 | 326 | ko05166 | 269 |
| 43 | ko00400 | 84 | 114 | ko00940 | 242 | 185 | ko04113 | 192 | 256 | ko04730 | 36 | 327 | ko05168 | 213 |
| 44 | ko00401 | 12 | 115 | ko00941 | 45 | 186 | ko04114 | 274 | 257 | ko04740 | 34 | 328 | ko05169 | 471 |
| 45 | ko00410 | 105 | 116 | ko00942 | 36 | 187 | ko04115 | 96 | 258 | ko04742 | 4 | 329 | ko05200 | 216 |
| 46 | ko00430 | 21 | 117 | ko00943 | 3 | 188 | ko04120 | 333 | 259 | ko04744 | 32 | 330 | ko05202 | 122 |
| 47 | ko00440 | 11 | 118 | ko00944 | 7 | 189 | ko04122 | 23 | 260 | ko04745 | 31 | 331 | ko05203 | 379 |
| 48 | ko00450 | 63 | 119 | ko00945 | 61 | 190 | ko04130 | 67 | 261 | ko04750 | 58 | 332 | ko05204 | 100 |
| 49 | ko00460 | 89 | 120 | ko00950 | 63 | 191 | ko04139 | 117 | 262 | ko04810 | 195 | 333 | ko05205 | 151 |
| 50 | ko00471 | 10 | 121 | ko00960 | 58 | 192 | ko04140 | 88 | 263 | ko04910 | 257 | 334 | ko05206 | 220 |
| 51 | ko00480 | 174 | 122 | ko00965 | 2 | 193 | ko04141 | 568 | 264 | ko04911 | 8 | 335 | ko05210 | 70 |
| 52 | ko00500 | 502 | 123 | ko00966 | 17 | 194 | ko04142 | 212 | 265 | ko04912 | 89 | 336 | ko05211 | 63 |
| 53 | ko00510 | 90 | 124 | ko00970 | 127 | 195 | ko04144 | 454 | 266 | ko04913 | 70 | 337 | ko05212 | 51 |
| 54 | ko00511 | 67 | 125 | ko00980 | 102 | 196 | ko04145 | 241 | 267 | ko04914 | 142 | 338 | ko05213 | 47 |
| 55 | ko00513 | 70 | 126 | ko00982 | 94 | 197 | ko04146 | 225 | 268 | ko04915 | 186 | 339 | ko05214 | 77 |
| 56 | ko00514 | 10 | 127 | ko00983 | 36 | 198 | ko04150 | 175 | 269 | ko04916 | 71 | 340 | ko05215 | 135 |
| 57 | ko00520 | 239 | 128 | ko01040 | 147 | 199 | ko04151 | 318 | 270 | ko04917 | 91 | 341 | ko05216 | 19 |
| 58 | ko00521 | 22 | 129 | ko01051 | 6 | 200 | ko04152 | 244 | 271 | ko04918 | 67 | 342 | ko05217 | 14 |
| 59 | ko00523 | 4 | 130 | ko01053 | 3 | 201 | ko04210 | 138 | 272 | ko04919 | 127 | 343 | ko05218 | 32 |
| 60 | ko00524 | 8 | 131 | ko01100 | 4374 | 202 | ko04211 | 138 | 273 | ko04920 | 121 | 344 | ko05219 | 22 |
| 61 | ko00531 | 44 | 132 | ko01110 | 2316 | 203 | ko04212 | 163 | 274 | ko04921 | 162 | 345 | ko05220 | 32 |
| 62 | ko00532 | 3 | 133 | ko01120 | 910 | 204 | ko04213 | 196 | 275 | ko04922 | 190 | 346 | ko05221 | 36 |
| 63 | ko00534 | 4 | 134 | ko01130 | 1036 | 205 | ko04214 | 91 | 276 | ko04923 | 23 | 347 | ko05222 | 38 |
| 64 | ko00540 | 16 | 135 | ko01200 | 685 | 206 | ko04215 | 13 | 277 | ko04924 | 56 | 348 | ko05223 | 26 |
| 65 | ko00550 | 1 | 136 | ko01210 | 132 | 207 | ko04260 | 70 | 278 | ko04925 | 31 | 349 | ko05230 | 127 |
| 66 | ko00561 | 161 | 137 | ko01212 | 284 | 208 | ko04261 | 129 | 279 | ko04930 | 76 | 350 | ko05231 | 158 |
| 67 | ko00562 | 143 | 138 | ko01220 | 19 | 209 | ko04270 | 75 | 280 | ko04931 | 147 | 351 | ko05322 | 122 |
| 68 | ko00563 | 32 | 139 | ko01230 | 538 | 210 | ko04310 | 180 | 281 | ko04932 | 259 | 352 | ko05323 | 72 |
| 69 | ko00564 | 179 | 140 | ko01502 | 1 | 211 | ko04320 | 34 | 282 | ko04933 | 52 | 353 | ko05340 | 2 |
| 70 | ko00565 | 63 | 141 | ko01503 | 1 | 212 | ko04330 | 35 | 283 | ko04940 | 30 | 354 | ko05410 | 40 |
| 71 | ko00590 | 42 | 142 | ko01521 | 85 | 213 | ko04340 | 67 | 284 | ko04960 | 16 | 355 | ko05416 | 40 |

**Supplementary Table S4**: Gene IDs list of clusters 8 and 11 of differentially expressed genes in four inflorescence tissue of ajowan

| **Cluster 8** | | | |  |  | **Cluster 11** | | |
| --- | --- | --- | --- | --- | --- | --- | --- | --- |
| **No.** | **Gene ID** | **No.** | **Gene ID** |  | **No.** | **Gene ID** |  |  |
| 1 | 56932_0_1 | 58 | 56279_2_1 |  | 1 | 11446_0_2 |  |  |
| 2 | 28120_0_1 | 59 | 30620_0_1 |  | 2 | 49273_0_2 |  |  |
| 3 | 30892_0_2 | 60 | 1606_0_2 |  | 3 | 48463_2_1 |  |  |
| 4 | 66939_0_1 | 61 | 52389_0_2 |  | 4 | 43393_0_1 |  |  |
| 5 | 29437_0_2 | 62 | 54371_0_1 |  | 5 | 16890_0_1 |  |  |
| 6 | 38875_0_1 | 63 | 53586_0_1 |  | 6 | 83905_0_1 |  |  |
| 7 | 2083_0_1 | 64 | 45131_0_2 |  | 7 | 52512_1_6 |  |  |
| 8 | 10745_0_1 | 65 | 35762_0_2 |  | 8 | 37784_0_2 |  |  |
| 9 | 43756_0_1 | 66 | 31945_0_3 |  | 9 | 10977_0_2 |  |  |
| 10 | 56570_0_2 | 67 | 54041_2_1 |  | 10 | 46179_0_1 |  |  |
| 11 | 54475_0_1 |  |  |  | 11 | 41501_0_2 |  |  |
| 12 | 57237_0_1 |  |  |  | 12 | 28584_0_2 |  |  |
| 13 | 17643_0_2 |  |  |  | 13 | 39301_0_1 |  |  |
| 14 | 56250_2_3 |  |  |  | 14 | 52880_0_3 |  |  |
| 15 | 12577_0_2 |  |  |  | 15 | 57095_0_1 |  |  |
| 16 | 55331_0_6 |  |  |  |  |  |  |  |
| 17 | 41958_0_3 |  |  |  |  |  |  |  |
| 18 | 56956_0_1 |  |  |  |  |  |  |  |
| 19 | 35243_0_1 |  |  |  |  |  |  |  |
| 20 | 52753_1_1 |  |  |  |  |  |  |  |
| 21 | 42362_0_2 |  |  |  |  |  |  |  |
| 22 | 56925_0_2 |  |  |  |  |  |  |  |
| 23 | 25410_0_2 |  |  |  |  |  |  |  |
| 24 | 51709_1_1 |  |  |  |  |  |  |  |
| 25 | 38280_0_1 |  |  |  |  |  |  |  |
| 26 | 8459_0_1 |  |  |  |  |  |  |  |
| 27 | 44969_0_1 |  |  |  |  |  |  |  |
| 28 | 37976_0_1 |  |  |  |  |  |  |  |
| 29 | 52229_0_1 |  |  |  |  |  |  |  |
| 30 | 46124_0_2 |  |  |  |  |  |  |  |
| 31 | 56792_3_5 |  |  |  |  |  |  |  |
| 32 | 48666_0_2 |  |  |  |  |  |  |  |
| 33 | 16796_0_1 |  |  |  |  |  |  |  |
| 34 | 43984_0_5 |  |  |  |  |  |  |  |
| 35 | 17119_0_2 |  |  |  |  |  |  |  |
| 36 | 33596_0_1 |  |  |  |  |  |  |  |
| 37 | 16684_0_2 |  |  |  |  |  |  |  |
| 38 | 51953_0_2 |  |  |  |  |  |  |  |
| 39 | 43662_0_1 |  |  |  |  |  |  |  |
| 40 | 6440_0_1 |  |  |  |  |  |  |  |
| 41 | 33664_0_1 |  |  |  |  |  |  |  |
| 42 | 52512_1_5 |  |  |  |  |  |  |  |
| 43 | 56932_0_3 |  |  |  |  |  |  |  |
| 44 | 55288_0_1 |  |  |  |  |  |  |  |
| 45 | 15786_0_1 |  |  |  |  |  |  |  |
| 46 | 11590_0_1 |  |  |  |  |  |  |  |
| 47 | 17342_0_1 |  |  |  |  |  |  |  |
| 48 | 56043_1_1 |  |  |  |  |  |  |  |
| 49 | 46103_0_3 |  |  |  |  |  |  |  |
| 50 | 43152_0_1 |  |  |  |  |  |  |  |
| 51 | 55315_0_1 |  |  |  |  |  |  |  |
| 52 | 49454_1_1 |  |  |  |  |  |  |  |
| 53 | 53964_0_2 |  |  |  |  |  |  |  |
| 54 | 68012_0_1 |  |  |  |  |  |  |  |
| 55 | 38176_0_1 |  |  |  |  |  |  |  |
| 56 | 55990_1_2 |  |  |  |  |  |  |  |
| 57 | 56455_2_2 |  |  |  |  |  |  |  |

**Supplementary Table S5**: Gene IDs list of clusters 5 and 15 of differentially expressed genes in four inflorescence tissue of ajowan

| **Cluster 5** | | | |  |  | **Cluster 15** | | |
| --- | --- | --- | --- | --- | --- | --- | --- | --- |
| **No.** | **Gene ID** | **No.** | **Gene ID** |  | **No.** | **Gene ID** |  |  |
| 1 | 45011_0_3 | 58 | 17460_0_3 |  | 1 | 28584_0_1 |  |  |
| 2 | 46179_0_3 | 59 | 22797_0_1 |  | 2 | 83905_0_2 |  |  |
| 3 | 29665_0_1 | 60 | 12577_0_1 |  | 3 | 48871_0_2 |  |  |
| 4 | 54349_0_1 | 61 | 56340_1_2 |  | 4 | 46160_0_1 |  |  |
| 5 | 55331_0_10 | 62 | 43572_1_2 |  | 5 | 19415_0_1 |  |  |
| 6 | 57085_0_1 | 63 | 39519_0_1 |  | 6 | 76698_0_1 |  |  |
| 7 | 49331_0_1 | 64 | 30582_0_2 |  | 7 | 35306_0_2 |  |  |
| 8 | 40326_0_2 | 65 | 56342_0_4 |  | 8 | 48127_0_1 |  |  |
| 9 | 29675_0_2 | 66 | 49016_2_1 |  | 9 | 17119_0_1 |  |  |
| 10 | 34896_0_3 | 67 | 34991_0_1 |  |  |  |  |  |
| 11 | 49188_0_1 | 68 | 34166_0_1 |  |  |  |  |  |
| 12 | 46744_0_1 | 69 | 31460_0_1 |  |  |  |  |  |
| 13 | 52586_0_2 | 70 | 44141_0_1 |  |  |  |  |  |
| 14 | 29578_0_2 | 71 | 35085_0_1 |  |  |  |  |  |
| 15 | 56366_0_1 | 72 | 39766_0_1 |  |  |  |  |  |
| 16 | 42681_1_1 | 73 | 51584_0_1 |  |  |  |  |  |
| 17 | 56964_0_3 | 74 | 42053_0_1 |  |  |  |  |  |
| 18 | 12072_0_1 | 75 | 54490_0_1 |  |  |  |  |  |
| 19 | 47963_0_2 | 76 | 56953_3_10 |  |  |  |  |  |
| 20 | 56104_0_6 | 77 | 26452_0_1 |  |  |  |  |  |
| 21 | 84365_0_1 | 78 | 47076_0_1 |  |  |  |  |  |
| 22 | 55787_1_1 | 79 | 36628_0_1 |  |  |  |  |  |
| 23 | 49211_0_1 | 80 | 16702_0_1 |  |  |  |  |  |
| 24 | 22797_0_2 | 81 | 28123_0_1 |  |  |  |  |  |
| 25 | 15752_0_1 | 82 | 22924_0_1 |  |  |  |  |  |
| 26 | 40755_0_1 | 83 | 42555_0_1 |  |  |  |  |  |
| 27 | 53507_0_2 | 84 | 53643_1_1 |  |  |  |  |  |
| 28 | 49313_0_1 | 85 | 37509_0_2 |  |  |  |  |  |
| 29 | 54041_2_2 | 86 | 31508_0_4 |  |  |  |  |  |
| 30 | 49615_0_1 | 87 | 50702_0_1 |  |  |  |  |  |
| 31 | 56442_0_1 | 88 | 55990_1_3 |  |  |  |  |  |
| 32 | 54094_0_3 | 89 | 28226_0_1 |  |  |  |  |  |
| 33 | 33006_0_2 | 90 | 54883_1_1 |  |  |  |  |  |
| 34 | 20029_0_1 | 91 | 103006_0_1 |  |  |  |  |  |
| 35 | 49033_1_2 | 92 | 56135_1_3 |  |  |  |  |  |
| 36 | 54475_0_3 | 93 | 33488_0_3 |  |  |  |  |  |
| 37 | 92277_0_1 | 94 | 44841_0_32 |  |  |  |  |  |
| 38 | 47682_0_1 | 95 | 56571_1_2 |  |  |  |  |  |
| 39 | 57031_1_2 | 96 | 19115_0_2 |  |  |  |  |  |
| 40 | 53134_0_1 | 97 | 43511_0_2 |  |  |  |  |  |
| 41 | 53413_1_3 | 98 | 54725_0_1 |  |  |  |  |  |
| 42 | 12262_0_2 | 99 | 51142_3_2 |  |  |  |  |  |
| 43 | 28258_0_2 | 100 | 56442_1_2 |  |  |  |  |  |
| 44 | 56250_1_1 | 101 | 61230_0_1 |  |  |  |  |  |
| 45 | 100428_0_1 | 102 | 50702_0_2 |  |  |  |  |  |
| 46 | 39259_0_2 | 103 | 31986_0_1 |  |  |  |  |  |
| 47 | 35278_0_3 | 104 | 49799_0_2 |  |  |  |  |  |
| 48 | 46437_0_1 | 105 | 6018_0_5 |  |  |  |  |  |
| 49 | 47661_0_2 | 106 | 37453_0_3 |  |  |  |  |  |
| 50 | 56904_1_1 | 107 | 55787_1_2 |  |  |  |  |  |
| 51 | 30768_0_1 | 108 | 726_0_1 |  |  |  |  |  |
| 52 | 12683_0_1 | 109 | 56342_0_2 |  |  |  |  |  |
| 53 | 50702_0_4 |  |  |  |  |  |  |  |
| 54 | 37518_1_2 |  |  |  |  |  |  |  |
| 55 | 35254_0_1 |  |  |  |  |  |  |  |
| 56 | 38437_0_1 |  |  |  |  |  |  |  |
| 57 | 50319_0_1 |  |  |  |  |  |  |  |

**Supplementary Table S6:** Sub categories of **GO:0019748** (secondary metabolic process) in biological process ontology type of differentially expressed genes using GO annotation by WEGO.

| **N** | **Term ID** | **Description** | **Gene ID** | **N** | **Term ID** | **Description** | **Gene ID** |
| --- | --- | --- | --- | --- | --- | --- | --- |
| 1 | GO:0006721 | terpenoid metabolic process | 20029  22727  24185  34607  36417  40842  41222  45011  45268  45584  49188  50434  56925  66029  91376 | 17 | GO:0016114 | terpenoid biosynthetic process | 20029  24185  34607  36417  40842  41222  45011  45584  49188  50434  56925  66029  91376 |
| 2 | GO:0033385 | geranylgeranyl diphosphate metabolic process | 91376 | 18 | GO:0016109 | tetraterpenoid biosynthetic process | 41222  49188  50434  56925  91376 |
| 3 | GO:0033386 | geranylgeranyl diphosphate biosynthetic process | 91376 | 19 | GO:0016117 | carotenoid biosynthetic process | 41222  49188  50434  56925  91376 |
| 4 | GO:0016098 | monoterpenoid metabolic process | 20029  36417  66029 | 20 | GO:0016123 | xanthophyll biosynthetic process | 56925 |
| 5 | GO:0016099 | monoterpenoid biosynthetic process | 20029  36417  66029 | 21 | GO:0016120 | carotene biosynthetic process | 66135 |
| 6 | GO:0006714 | sesquiterpenoid metabolic process | 45011  56925 | 22 | GO:0033383 | geranyl diphosphate metabolic process | 91376 |
| 7 | GO:0016106 | sesquiterpenoid biosynthetic process | 45011  56925 | 23 | GO:0033384 | geranyl diphosphate biosynthetic process | 91376 |
| 8 | GO:0009688 | abscisic acid biosynthetic process | 56925 | 24 | GO:0016108 | tetraterpenoid metabolic process | 22727  41222  49188  50434  56925  91376 |
| 9 | GO:0009687 | abscisic acid metabolic process | 56925 | 25 | GO:0016116 | carotenoid metabolic process | 22727  41222  49188  50434  56925  91376 |
| 10 | GO:0043288 | apocarotenoid metabolic process | 56925 | 26 | GO:0016122 | xanthophyll metabolic process | 22727  56925 |
| 11 | GO:0043289 | apocarotenoid biosynthetic process | 56925 | 27 | GO:0010028 | xanthophyll cycle | 22727 |
| 12 | GO:0009687 | abscisic acid metabolic process | 56925 | 28 | GO:0016119 | carotene metabolic process | 66135 |
| 13 | GO:0016101 | diterpenoid metabolic process | 40842  45268  45584 | 29 | GO:0016103 | diterpenoid catabolic process | 45268 |
| 14 | GO:0009685 | gibberellin metabolic process | 40842  45268  45584 | 30 | GO:0016102 | diterpenoid biosynthetic process | 40842  45584 |
| 15 | GO:0045487 | gibberellin catabolic process | 45268 | 31 | GO:0016115 | terpenoid catabolic process | 45268 |
| 16 | GO:0009686 | gibberellin biosynthetic process | 40842  45584 |

**Supplementary Table S7:** GO enrichment analysis for DEGs unigenes related to the terpenoid process, represented in comparison of four genotypes.

| **Sets** | **category** | **over represented pvalue** | **under represented pvalue** | **Number of DE In category** | **Number In category** | **over represented FDR** | **go_term** |
| --- | --- | --- | --- | --- | --- | --- | --- |
| Arak10 vs Shiraz17 | GO:0016109 | 0.015344038 | 0.998166367 | 3 | 49 | 1 | tetraterpenoid biosynthetic process |
| Arak10 vs Shiraz17 | GO:0016108 | 0.02468951 | 0.99644908 | 3 | 59 | 1 | tetraterpenoid metabolic process |
| Arak10 vs Shiraz21 | GO:0046246 | 0.00086876 | 0.99998936 | 2 | 13 | 1 | terpene biosynthetic process |
| Arak10 vs Shiraz21 | GO:0042214 | 0.00472262 | 0.999852517 | 2 | 30 | 1 | terpene metabolic process |
| Arak3 vs Shiraz17 | GO:0016114 | 0.002800076 | 0.999585077 | 5 | 290 | 1 | terpenoid biosynthetic process |
| Arak3 vs Shiraz17 | GO:0006721 | 0.005091123 | 0.999131633 | 5 | 334 | 1 | terpenoid metabolic process |
| Arak3 vs Shiraz17 | GO:0016109 | 0.010805497 | 0.999472185 | 2 | 49 | 1 | tetraterpenoid biosynthetic process |
| Arak3 vs Shiraz17 | GO:0016108 | 0.015231572 | 0.99910512 | 2 | 59 | 1 | tetraterpenoid metabolic process |
| Arak3 vs Shiraz17 | GO:0016106 | 0.016196568 | 0.999017225 | 2 | 59 | 1 | sesquiterpenoid biosynthetic process |
| Arak3 vs Shiraz17 | GO:0006714 | 0.02442399 | 0.998149702 | 2 | 73 | 1 | sesquiterpenoid metabolic process |
| Arak3 vs Shiraz21 | GO:0016103 | 0.005400866 | 0.999992756 | 1 | 2 | 1 | diterpenoid catabolic process |
| Arak3 vs Shiraz21 | GO:0016101 | 0.024245078 | 0.998168721 | 2 | 88 | 1 | diterpenoid metabolic process |

**Supplementary Table S8:** Unigenes encoding enzymes involved in terpenoid biosynthesis in *T.ammi*

| Gene | KEGG Pathway | KEGG Entry | Enzyme | Enzyme Class | Reaction | Definition | Unigene id |
| --- | --- | --- | --- | --- | --- | --- | --- |
| AACT | ko00900 | K00626 | 2.3.1.9 | Transferases | R00238 | acetyl-CoA C-acetyltransferase | 41178_0_1 45391_0_1 16455_0_1 92691_0_1 38901_0_1 25492_0_1 25492_0_2 51784_0_2 57396_0_1 59486_0_1 42255_1_1 |
| HMGS | ko00900 | K01641 | 2.3.3.10 | Transferases | R01978 | hydroxymethylglutaryl-CoA synthase | 43372_0_1 |
| HMGR | ko00900 | K00021 | 1.1.1.34 | Oxidoreductases | R02082 | hydroxymethylglutaryl-CoA reductase | 100091_0_1 21724_0_1 87803_0_1 43134_0_1 879_0_1 17049_0_1 17049_0_2 46370_0_1 |
| MK | ko00900 | K00869 | 2.7.1.36 | Transferases | R02245 | mevalonate kinase | 54851_0_1 28766_0_1 23196_0_1 |
| PMK | ko00900 | K00938 | 2.7.4.2 | Transferases | R03245 | phosphomevalonate kinase | 56695_0_1  56695_0_2  56695_2_1 89833_0_1 |
| MDD | ko00900 | K01597 | 4.1.1.33 | Lyases | R01121 | diphosphomevalonate decarboxylase | 104780_0_1 43442_0_1 |
| IPK | ko00900 | K06981 | 2.7.4.26 | Transferases | R10093 | isopentenyl phosphate kinase | 54877_1_1 |
| DXS | ko00900 | K01662 | 2.2.1.7 | Transferases | R05636 | 1-deoxy-D-xylulose-5-phosphate synthase | 44346_0_1 46265_0_1 836_0_1 50586_0_1 82996_0_1 30805_0_1 |
| DXR | ko00900 | K00099 | 1.1.1.267 | Oxidoreductases | R05688 | 1-deoxy-D-xylulose-5-phosphate reductoisomerase | 44875_0_1 93434_0_1 |
| CDP-MES | ko00900 | K00991 | 2.7.7.60 | Transferases | R05633 | 2-C-methyl-D-erythritol 4-phosphate cytidylyltransferase | 42987_0_1 |
| CDP-MEK | ko00900 | K00919 | 2.7.1.148 | Transferases | R05634 | 4-diphosphocytidyl-2-C-methyl-D-erythritol kinase | 36615_0_1 |
| MECPS | ko00900 | K01770 | 4.6.1.12 | Lyases | R05637 | 2-C-methyl-D-erythritol 2,4-cyclodiphosphate synthase | 37821_0_1 |

Continued

| Gene | KEGG Pathway | KEGG Entry | Enzyme | Enzyme Class | Reaction | Definition | Unigene id |
| --- | --- | --- | --- | --- | --- | --- | --- |
| HDS | ko00900 | K03526 | 1.17.7.1 1.17.7.3 | Oxidoreductases | R08689  R10859 | (E)-4-hydroxy-3-methylbut-2-enyl-diphosphate synthase | 47244_1_1 21529_0_1 |
| HDR | ko00900 | K03527 | 1.17.7.4 | Oxidoreductases | R05884  R08210 | 4-hydroxy-3-methylbut-2-en-1-yl diphosphate reductase | 56344_0_1 9671_0_1 52395_0_1 29238_0_1 57675_0_1 51891_1_1 39578_0_1 37220_0_1 96096_0_1 |
| IDI | ko00900 | K01823 | 5.3.3.2 | Isomerases | R01123 | isopentenyl-diphosphate Delta-isomerase | 41437_0_1 |
| GDS | ko00900 | K14066 | 2.5.1.1 | Transferases | R01658 | geranyl diphosphate synthase | 35063_0_1 35063_0_2 |
| FDS | ko00900 | K15887 | 2.5.1.92 | Transferases | R09633 | (2Z,6Z)-farnesyl diphosphate synthase | 90713_0_1 |
| *ta*_TPS1 | ko00902 | K18108 | 4.2.3.111 | Lyases | R06421 | (-)-alpha-terpineol synthase | 61038_0_1 37637_0_1 87322_0_1 19758_0_1 19758_0_2 |
| *ta*_TPS2 | ko00902 | K15096 | 4.2.3.20 | Lyases | R06120 | (R)-limonene synthase | 45993_0_1 45993_0_2 44646_0_1 56475_0_1 98518_0_1  40869_0_1  16174_0_2  39518_0_1 |
| *ta*_TPS3 | ko00902 | K12467 | 4.2.3.15 | Lyases | R02009 | myrcene/ocimene synthase | 94123_0_1 45993_1_1 88584_0_1 31054_0_1 |
| *ta*_DH | ko00902 | K15090 | 1.1.1.223 | Oxidoreductases | R03261 | isopiperitenol dehydrogenase | 55232_0_1 66029_0_1 |
| *ta*_RD | ko00902 | K15092 | 1.3.1.81 | Oxidoreductases | R06419  R06420 | **(**+**)**-pulegone reductase | 25626_0_1 |
| *ta*_DH | ko00902 | K15095 | 1.1.1.208 | Oxidoreductases | R02548 | (+)-neomenthol dehydrogenase | 51174_0_2 62192_0_1 52551_0_1 |

Continued

| Gene | KEGG Pathway | KEGG Entry | Enzyme | Enzyme Class | Reaction | Definition | Unigene id |
| --- | --- | --- | --- | --- | --- | --- | --- |
| CYP76B6 | ko00902 | K15099 | 1.14.13.152 | Oxidoreductases | R08785 | geraniol 8-hydroxylase | 104490_0_1 104490_0_2 78967_0_1 20029_0_1 20029_0_2 56095_0_1 56095_0_2 56095_1_1 72499_0_1 36417_0_1 74139_0_1  74139_0_2  74139_0_3 |
| CYP72A1 | ko00902 | K13400 | 1.3.3.9 | Oxidoreductases | R05833 | secologanin synthase | 56451_0_2  51607_0_1 74713_0_1 49536_0_3 |
| GGPPS | ko00900 | K13789 | 2.5.1.29 | Transferases | R02061 | geranylgeranyl diphosphate synthase | 44310_0_1  30279_0_1 91376_0_1 74796_0_1 42603_0_1 28035_0_1 |
| KS | ko00904 | K04120 | 5.5.1.13 | Isomerases | R02068 | ent-copalyl diphosphate/ent-kaurene synthase | 39849_0_1 |
| KS | ko00904 | K04121 | 4.2.3.19 | Lyases | R05092 | ent-kaurene synthase | 43181_0_2 16331_0_1 71091_0_1 49081_0_1 6602_0_1 76743_0_1 |
| GA3 | ko00904 | K04122 | 1.14.13.78 | Oxidoreductases | R06291  R06292  R06293 | ent-kaurene oxidase | 100198_0_1 103706_0_1 41965_0_1 3752_0_1 3752_0_2 63493_0_1 |
| KAO | ko00904 | K04123 | 1.14.13.79 | Oxidoreductases | R06294  R06295  R06296  R06297 | ent-kaurenoic acid hydroxylase | 11786_0_1 11786_0_2 61777_0_1 46645_0_1 40842_0_1 |
| GA20OX | ko00904 | K05282 | 1.14.11.12 | Oxidoreductases | R06322 R06323 R06326 | gibberellin 20-oxidase | 66821_0_1 53543_0_1 48022_0_1 84973_0_1 80074_0_1 5921_0_1 5921_0_2 26098_0_1 44006_0_1 |

Continued

| Gene | KEGG Pathway | KEGG Entry | Enzyme | Enzyme Class | Reaction | Definition | Unigene id |
| --- | --- | --- | --- | --- | --- | --- | --- |
| GA2OX | ko00904 | K04125 | 1.14.11.13 | Oxidoreductases | R06337 R06338 | gibberellin 2-oxidase | 105318_0_1 105713_0_1 50337_0_1 50337_0_2  50337_0_3 56758_0_2 84036_0_1 93307_0_1 43192_0_1 42893_0_1 16384_0_1 54559_2_1 84941_0_1 84941_0_2 71167_0_1 66924_0_1 17510_0_1 3646_0_1 |
| GA3OX | ko00904 | K04124 | 1.14.11.15 | Oxidoreductases | R06336 | gibberellin 3-beta-dioxygenase | 43399_0_1 39640_0_1 59363_0_1 98261_0_1 45584_0_1 |
| FDFT1 | ko00909 | K00801 | 2.5.1.21 | Transferases | R00702 R02872 | farnesyl-diphosphate farnesyltransferase | 40037_0_1 |
| SQLE | ko00909 | K00511 | 1.14.14.17 | Oxidoreductases | R02874 | squalene monooxygenase | 44174_0_1  53819_0_1 54305_0_1 54305_0_2 54305_1_1 54305_1_2 49111_0_1 |
| LUP2 | ko00909 | K15813 | 5.4.99.39 | Isomerases | R06469 | alpha/beta-amyrin synthase | 77323_0_1 51672_0_1 60789_0_1 31938_0_1 31938_0_2 31938_1_1 5492_0_1 59054_0_1 |
| DDS | ko00909 | K15817 | 4.2.1.125 | Lyases | R09701 | dammarenediol II synthase | 51672_0_1 |
| AFS1 | ko00909 | K14173 | 4.2.3.46 | Lyases | R08696 | alpha-farnesene synthase | 102906_0_1 58740_0_1 |

**Supplementary Table S9:** Differentially expressed and annotation of total unigenes of gene families members which involved in terpenoid biosynthesis in *T.ammi*.

| **Gene Family** | **Total**  **unigenes** | **Carrot** | **TAIR10** | ***P. quinquefolius*** | **Differentially**  **expressed** |
| --- | --- | --- | --- | --- | --- |
| Cytochrome P450 (CYP450) | 203 | 203 | 203 | 203 | 25 |
| Terpene synthase (TPS) | 38 | 38 | 38 | 38 | 4 |
| Dehydrogenase (DH) | 1230 | 1125 | 1116 | 1073 | 53 |
| Transcription factor (TF) | 1831 | 1070 | 113 | 1797 | 73 |

**Supplementary Table S10:** Number of unitranscripts for each transcription factor genes families of *T. ammi*

| **Percent** | **No. of Unitranscripts** | **TF families** |
| --- | --- | --- |
| 9.63 | 249 | bHLH |
| 6.88 | 178 | NAC |
| 5.57 | 144 | MYB_related |
| 5.53 | 143 | C2H2 |
| 5.41 | 140 | ERF |
| 4.83 | 125 | bZIP |
| 4.56 | 118 | MYB |
| 3.36 | 87 | C3H |
| 3.36 | 87 | Trihelix |
| 3.36 | 87 | WRKY |
| 3.25 | 84 | HD-ZIP |
| 3.02 | 78 | G2-like |
| 3.02 | 78 | GRAS |
| 2.86 | 74 | FAR1 |
| 2.71 | 70 | GATA |
| 2.36 | 61 | B3 |
| 2.20 | 57 | ARF |
| 2.17 | 56 | TALE |
| 1.86 | 48 | LBD |
| 1.70 | 44 | TCP |
| 1.39 | 36 | SBP |
| 1.35 | 35 | AP2 |
| 1.20 | 31 | CO-like |
| 1.16 | 30 | HB-other |
| 1.16 | 30 | NF-YB |
| 1.01 | 26 | MIKC_MADS |
| 1.01 | 26 | Nin-like |
| 1.01 | 26 | YABBY |
| 0.93 | 24 | Dof |
| 0.93 | 24 | HSF |
| 0.89 | 23 | NF-YC |
| 0.85 | 22 | CPP |
| 0.77 | 20 | E2F/DP |
| 0.77 | 20 | SRS |
| 0.77 | 20 | WOX |
| 0.73 | 19 | ARR-B |
| 0.73 | 19 | GRF |
| 0.66 | 17 | BES1 |
| 0.62 | 16 | BBR-BPC |
| 0.58 | 15 | CAMTA |
| 0.54 | 14 | NF-YA |
| 0.43 | 11 | GeBP |
| 0.43 | 11 | LSD |
| 0.43 | 11 | STAT |
| 0.35 | 9 | DBB |
| 0.31 | 8 | M-type_MADS |
| 0.23 | 6 | NF-X1 |
| 0.19 | 5 | ZF-HD |
| 0.15 | 4 | RAV |
| 0.15 | 4 | S1Fa-like |
| 0.15 | 4 | VOZ |
| 0.15 | 4 | Whirly |
| 0.12 | 3 | EIL |
| 0.12 | 3 | HRT-like |
| 0.04 | 1 | HB-PHD |
| 0.04 | 1 | SAP |
|  | **2586** | **Total** |

**Supplementary Table S11:** Homology of TFs genes with plant species

| **Species** | **No. of Unitranscripts** | **Percent** | **Species** | **No. of Unitranscripts** | **Percent** |
| --- | --- | --- | --- | --- | --- |
| Daucus carota | 1422 | 54.92 | Sesamum indicum | 5 | 0.19 |
| Actinidia chinensis | 115 | 4.44 | Brassica oleracea | 4 | 0.15 |
| Malus domestica | 102 | 3.94 | Cicer arietinum | 4 | 0.15 |
| Oryza longistaminata | 50 | 1.93 | Dianthus caryophyllus | 4 | 0.15 |
| Fragaria x ananassa | 45 | 1.74 | Gossypium arboreum | 4 | 0.15 |
| Musa acuminata | 39 | 1.51 | Gossypium raimondii | 4 | 0.15 |
| Brassica napus | 37 | 1.43 | Hordeum vulgare | 4 | 0.15 |
| Fragaria vesca | 30 | 1.16 | Nicotiana tomentosiformis | 4 | 0.15 |
| Gossypium hirsutum | 28 | 1.08 | Oryza nivara | 4 | 0.15 |
| Populus euphratica | 28 | 1.08 | Pyrus bretschneideri | 4 | 0.15 |
| Ipomoea trifida | 27 | 1.04 | Salix purpurea | 4 | 0.15 |
| Sisymbrium irio | 26 | 1.00 | Utricularia gibba | 4 | 0.15 |
| Phyllostachys heterocycla | 25 | 0.97 | Vigna radiata | 4 | 0.15 |
| Jatropha curcas | 24 | 0.93 | Amborella trichopoda | 3 | 0.12 |
| Aegilops tauschii | 23 | 0.89 | Arabidopsis halleri | 3 | 0.12 |
| Raphanus raphanistrum | 23 | 0.89 | Arabidopsis lyrata | 3 | 0.12 |
| Linum usitatissimum | 22 | 0.85 | Dichanthelium oligosanthes | 3 | 0.12 |
| Nicotiana benthamiana | 20 | 0.77 | Juglans regia | 3 | 0.12 |
| Dorcoceras hygrometricum | 19 | 0.73 | Nelumbo nucifera | 3 | 0.12 |
| Solanum melongena | 19 | 0.73 | Oryza glumaepatula | 3 | 0.12 |
| Arachis ipaensis | 16 | 0.62 | Oryza sativa | 3 | 0.12 |
| Oryza meridionalis | 16 | 0.62 | Phalaenopsis equestris | 3 | 0.12 |
| Zoysia pacifica | 16 | 0.62 | Solanum tuberosum | 3 | 0.12 |
| Camelina sativa | 15 | 0.58 | Triticum aestivum | 3 | 0.12 |
| Carica papaya | 14 | 0.54 | Triticum urartu | 3 | 0.12 |
| Leersia perrieri | 14 | 0.54 | Aethionema arabicum | 2 | 0.08 |
| Raphanus sativus | 14 | 0.54 | Citrullus lanatus | 2 | 0.08 |
| Arachis duranensis | 13 | 0.50 | Mimulus guttatus | 2 | 0.08 |
| Petunia inflata | 12 | 0.46 | Petunia axillaris | 2 | 0.08 |
| Picea sitchensis | 12 | 0.46 | Populus trichocarpa | 2 | 0.08 |
| Prunus persica | 12 | 0.46 | Prunus mume | 2 | 0.08 |
| Ziziphus jujuba | 12 | 0.46 | Selaginella moellendorffii | 2 | 0.08 |
| Solanum pennellii | 11 | 0.42 | Setaria italica | 2 | 0.08 |
| Vitis vinifera | 11 | 0.42 | Spinacia oleracea | 2 | 0.08 |
| Azadirachta indica | 10 | 0.39 | Aquilegia coerulea | 1 | 0.04 |
| Oropetium thomaeum | 10 | 0.39 | Arachis hypogaea | 1 | 0.04 |
| Artemisia annua | 9 | 0.35 | Boechera stricta | 1 | 0.04 |
| Brachypodium distachyon | 9 | 0.35 | Cannabis sativa | 1 | 0.04 |
| Coffea canephora | 9 | 0.35 | Capsicum annuum | 1 | 0.04 |
| Manihot esculenta | 9 | 0.35 | Cucumis sativus | 1 | 0.04 |
| Salvia miltiorrhiza | 9 | 0.35 | Eragrostis tef | 1 | 0.04 |
| Glycine soja | 8 | 0.31 | Genlisea aurea | 1 | 0.04 |
| Nicotiana tabacum | 8 | 0.31 | Kalanchoe marnieriana | 1 | 0.04 |
| Catharanthus roseus | 7 | 0.27 | Lactuca sativa | 1 | 0.04 |
| Glycine max | 7 | 0.27 | Monoraphidium neglectum | 1 | 0.04 |
| Oryza punctata | 7 | 0.27 | Nicotiana sylvestris | 1 | 0.04 |
| Pseudotsuga menziesii | 7 | 0.27 | Oryza barthii | 1 | 0.04 |
| Medicago truncatula | 6 | 0.23 | Oryza glaberrima | 1 | 0.04 |
| Morus notabilis | 6 | 0.23 | Panicum virgatum | 1 | 0.04 |
| Solanum pimpinellifolium | 6 | 0.23 | Phaseolus vulgaris | 1 | 0.04 |
| Theobroma cacao | 6 | 0.23 | Spirodela polyrhiza | 1 | 0.04 |
| Trifolium pratense | 6 | 0.23 | Vigna angularis | 1 | 0.04 |
| Zea mays | 6 | 0.23 | Zoysia japonica | 1 | 0.04 |
| Ocimum tenuiflorum | 5 | 0.19 | Zoysia matrella | 1 | 0.04 |

**Supplementary Table S12:** List of different oligonucleotide primers of selected genes used for QRT-PCR

| NO. | Gene | Forward (5’-3’) | Reverse (5’-3’) |
| --- | --- | --- | --- |
| 1 | 56475 | TCCCTCTCAAGGAATCTAACCC | GTTTCATGTTATCAGGCACTCC |
| 2 | 19758 | ACAAGGCAGGCTACAATCCC | GCCATTTCTGACGAGACCAC |
| 3 | 37637 | GTTTGTAAGTTTCTTCACTGTCTTG | GTCGATAAATCCAGACCTTTCCC |
| 4 | 4086 | TGCCAAAAATTAGAGAACCCAG | TGAGCACACATAATCAGGGACA |
| 5 | SAND | TTGTAAGCTGAGTCTGTAATCCATC | CCTAAAGTGACCAGAAACACAAG |
| 6 | eIF-4a | CATGCGTGAGTTCCGTTCTG | GCAGCTCCTCGACAACCAC |

**Supplementary Table S13:** GenBank accession numbers of terpenoids genes identified in *T. ammi.*

| **NO.** | **Gene name** | **GenBank accession numbers** | **Gene length (bp)** | **Gene type** |
| --- | --- | --- | --- | --- |
| 1 | gamma-terpinene synthase (ta-GTS) | MG470839 | 1827 | complete cds |
| 2 | terpene synthase (TPS2) | MG470840 | 2226 | complete cds |
| 3 | terpene synthase (TPS1) | MG470841 | 2587 | complete cds |
| 4 | monoterpene synthase (TPS3) | MG470842 | 2065 | complete cds |
| 5 | cytochrome p450 (cyp71d500) | MG470838 | 1539 | complete cds |
| 6 | 1-deoxy-D-xylulose 5-phosphate reductoisomerase (DXR) | MG762013 | 2238 | complete cds |
| 7 | 1-deoxy-D-xylulose-5-phosphate synthase (DXS) | MG762014 | 3079 | complete cds |
| 8 | gibberellin 2-beta-dioxygenase (GA2ox) | MG762015 | 1430 | complete cds |
| 9 | ent-kaurene oxidase (KO) | MG762016 | 1997 | complete cds |
| 10 | gibberellin 3-beta-dioxygenase (GA3OX) | MG762017 | 1404 | complete cds |
| 11 | gibberellin 20 oxidase (GA20ox) | MG762018 | 1506 | complete cds |
| 12 | geranyl pyrophosphate synthase (GPS) | MG762019 | 1804 | partial cds |
| 13 | geranylgeranyl pyrophosphate synthase (GGPS), isoform X3 | MG762020 | 1469 | complete cds |
| 14 | heterodimeric geranylgeranyl pyrophosphate synthase small subunit, isoform X1 | MG762021 | 1891 | complete cds |
| 15 | geranylgeranyl pyrophosphate synthase (GGPS), isoform X2 | MG762022 | 1765 | complete cds |
| 16 | heterodimeric geranylgeranyl pyrophosphate synthase small subunit, isoform X2 | MG762023 | 1757 | complete cds |
| 17 | geranylgeranyl pyrophosphate synthase (GGPS), isoform X1 | MG762024 | 1504 | complete cds |
| 18 | 4-hydroxy-3-methylbut-2-enyl diphosphate reductase (HDR), isoform X1 | MG762025 | 1720 | complete cds |
| 19 | 4-hydroxy-3-methylbut-2-enyl diphosphate reductase (HDR), isoform X2 | MG762026 | 1659 | complete cds |
| 20 | 4-hydroxy-3-methylbut-2-enyl diphosphate reductase (HDR), isoform X3 | MG762027 | 1947 | complete cds |
| 21 | 4-hydroxy-3-methylbut-2-en-1-yl diphosphate synthase (ferredoxin) | MG762028 | 2808 | complete cds |
| 22 | ent-kaurenoic acid oxidase (KAO) | MG762029 | 1855 | complete cds |
| 23 | ent-kaurene synthase (KS), isoform X1 | MG762030 | 1708 | complete cds |
| 24 | ent-kaurene synthase (KS), isoform X2 | MG762031 | 1587 | complete cds |
| 25 | 2C-methyl-D-erythritol 2,4-cyclodiphosphate synthase (MECPS) | MG762032 | 1100 | partial cds |
| 26 | 4-diphosphocytidyl-2-C-methyl-Derythritol kinase (CDPMEK) | MG762033 | 1492 | partial cds |
| 27 | 2-C-methyl-D-erythritol4-phosphate cytidylyl transferase (CDPMES), isoform X1 | MG762034 | 1595 | complete cds |
| 28 | 2-C-methyl-D-erythritol4-phosphate cytidylyl transferase (CDPMES), isoform X2 | MG762035 | 1460 | complete cds |
| 29 | short-chain dehydrogenase/reductase 2b | MG762036 | 1462 | complete cds |
| 30 | acetyl-CoA C-acetyltransferase (AACT), isoform1 | MG745850 | 2001 | complete cds |
| 31 | acetyl-CoA C-acetyltransferase (AACT), isoform2 | MG745851 | 1706 | complete cds |
| 32 | hydroxymethylglutaryl-CoA synthase (HMGS) | MG745852 | 1995 | complete cds |
| 33 | hydroxy methylglutaryl CoA reductase (HMGR) | MG745853 | 1545 | partial cds |
| 34 | mevalonate kinase (MVK) | MG745854 | 1536 | complete cds |
| 35 | phosphomevalonate kinase (PMVK) | MG745855 | 1929 | complete cds |
| 36 | diphosphomevalonate decarboxylase (MVD) | MG745856 | 1855 | complete cds |
| 37 | isopentenyl-diphosphate Delta-isomerase (IDI) | MG745857 | 1325 | complete cds |
| 38 | farnesol kinase (FOLK) | MG745858 | 1180 | complete cds |
| 39 | farnesylcysteine lyase (FCLY) | MG745859 | 1910 | complete cds |
| 40 | prenylcysteine alpha-carboxyl methylesterase (PCME) | MG745860 | 1876 | complete cds |
| 41 | protein-S-isoprenylcysteine O-methyltransferase (ICMT) | MG745861 | 852 | complete cds |
| 42 | STE24 endopeptidase (STE24) | MG745862 | 1677 | complete cds |
| 43 | prenyl protein peptidase (FACE2) | MG745863 | 1102 | partial cds |
| 44 | protein farnesyltransferase subunit beta (FNTB) | MG745864 | 1772 | complete cds |
| 45 | ditrans,polycis-polyprenyl diphosphate synthase (DHDDS) | MG745865 | 1472 | complete cds |
| 46 | squalene synthase (SQS) | MG745866 | 1555 | complete cds |
| 47 | squalene monooxygenase (SQLE) | MG745867 | 2158 | complete cds |
| 48 | beta-amyrin synthase (LUP4) | MG745868 | 1833 | complete cds |
| 49 | dammarenediol II synthase (DDS) | MG745869 | 2162 | partial cds |
| 50 | alpha-farnesene synthase (AFS1) | MG745870 | 571 | internal cds |
| 51 | NAD+-dependent farnesol dehydrogenase (FLDH) | MG745872 | 1729 | complete cds |
| 52 | cytochrome p450 (cyp71d499) | MG518621 | 984 | partial cds |
| 53 | cytochrome p450 (cyp71d508) | MG518620 | 1536 | complete cds |
| 54 | cytochrome p450 (cyp71d509) | MG518619 | 1506 | complete cds |

**Supplementary Table S14**. List of identified terpene synthase unigenes of *T. ammi* and their respective associated sequence IDs and functional annotation in *P. quinquefolius*.

| **No.** | **Terpene Synthase Unigenes of *T. ammi*** | **Sequence ID in *P. quinquefolius*** | **Unified Functional Annotation of Sequence ID in *P. quinquefolius*** |
| --- | --- | --- | --- |
| 1 | 91969_0_1 | pqa_locus_10504_so_3_len_1847_ver_2 | Terpenesynthase-1 |
| 2 | 87322_0_1 | pqa_locus_11839_so_2_len_1099_ver_2 | Unknown |
| 3 | 89209_0_1 | pqa_locus_15525_so_1_len_1981_ver_2 | Unknown |
| 4 | 23343_0_1 |
| 5 | 1688_0_1 |
| 6 | 46667_1_1 |
| 7 | 11043_0_2 | pqa_locus_15525_so_4_len_2053_ver_2 | Unknown |
| 8 | 1688_0_2 | pqa_locus_15525_so_5_len_878_ver_2 | Unknown |
| 9 | 88584_0_1 |
| 10 | 11043_0_1 | pqa_locus_15525_so_6_len_1985_ver_2 | Unknown |
| 11 | 34607_0_1 |
| 12 | 46667_0_1 |
| 13 | 46667_0_2 |
| 14 | 102704_0_1 |
| 15 | 46691_0_1 |
| 16 | 40863_0_1 |
| 17 | 39849_0_1 | pqa_locus_16937_so_1_len_812_ver_2 | Copalyl diphosphate synthase |
| 18 | 76743_0_1 | pqa_locus_17011_so_4_len_1007_ver_2 | Unknown |
| 19 | 6602_0_1 | pqa_locus_17011_so_5_len_1865_ver_2 | Ent-kaurene synthase B, chloroplast |
| 20 | 49081_0_1 |
| 21 | 43181_0_2 | pqa_locus_23853_so_1_len_701_ver_2 | Ent-kaurene synthase No1 |
| 22 | 41406_0_1 | pqa_locus_25738_so_1_len_601_ver_2 | (R)-limonene synthase |
| 23 | 102906_0_1 | pqa_locus_30737_so_1_len_1535_ver_2 | (E)-beta-ocimene/(E,E)-alpha-farnesene synthase |
| 24 | 107170_0_1 | pqa_locus_45093_so_1_len_636_ver_2 | (3S)-linalool/(E)-nerolidol synthase |
| 25 | 45993_1_1 | pqa_locus_6352_so_1_len_1694_ver_2 | Unknown |
| 26 | 94902_0_1 |
| 27 | 98518_0_1 | pqa_locus_6352_so_3_len_1313_ver_2 | Unknown |
| 28 | 37637_0_1 | pqa_locus_6352_so_4_len_1838_ver_2 | Unknown |
| 29 | 19758_0_2 |
| 30 | 19758_0_1 |
| 31 | 45993_0_2 |
| 32 | 40869_0_1 |
| 33 | 44646_1_3 |
| 34 | 45993_0_1 |
| 35 | 56475_0_1 |
| 36 | 44646_1_2 |
| 37 | 50287_0_1 | pqa_locus_6352_so_5_len_2134_ver_2 | (+)-limonene synthase 2 |
| 38 | 86302_0_1 | pqa_locus_6352_so_6_len_2131_ver_2 | Unknown |

**Supplementary Table S15**. The description of identified genes of clusters 5,8,11 and 15 of differentially expressed genes in four inflorescence tissue of ajowan.

| **Cluster** | **Unigene ID** | **Gene symbol** | **Gene name** | **Uniprot_Entry** |
| --- | --- | --- | --- | --- |
| **Cluster 8** | 52512_1_5 | *RPS5* | *30S ribosomal protein S5, chloroplastic* | UPI0000135064 |
|  | 29437_0_2 | *HDS* | *4-hydroxy-3-methylbut-2-en-1-yl diphosphate synthase (ferredoxin), chloroplastic* | UPI0000666896 |
|  | 55990_1_2 | *dis2* | *Actin-related protein 2/3 complex subunit 2A* | UPI0000131FAB |
|  | 35243_0_1 | *MP* | *Auxin response factor 5* | UPI00004B459F |
|  | 43152_0_1 | *BGLU30* | *Beta-glucosidase 30* | UPI00000A14D4 |
|  | 46103_0_3 | *TENA_E* | *Bifunctional TENA-E protein* | UPI000009F32D |
|  | 54371_0_1 | *BOR4* | *Boron transporter 4* | UPI00000A8048 |
|  | 6440_0_1 | *CAMS1* | *Camelliol C synthase* | UPI0000163302 |
|  | 66939_0_1 | *COR413PM2* | *Cold-regulated 413 plasma membrane protein 2* | UPI00000A7460 |
|  | 12577_0_2 | *COX2* | *Cytochrome c oxidase subunit 2* | UPI000012800C |
|  | 42362_0_2 | *RMA2* | *E3 ubiquitin-protein ligase RMA2* | UPI000000BEB5 |
|  | 30620_0_1 | *At1g57790* | *F-box/kelch-repeat protein At1g57790* | UPI00000A9031 |
|  | 38875_0_1 | *GSTT3* | *Glutathione S-transferase T3* | UPI0000048BE6 |
|  | 17643_0_2 | *MAP2B* | *Methionine aminopeptidase 2B* | UPI000005EC03 |
|  | 16684_0_2 | *MLO-H1* | *MLO-like protein 1* | UPI000003094F |
|  | 53586_0_1 | *ND6* | *NADH-ubiquinone oxidoreductase chain 6* | UPI0000130892 |
|  | 43662_0_1 | *PAE8* | *Pectin acetylesterase 8* | UPI0000162AC7 |
|  | 2083_0_1 | *PGMP* | *Phosphoglucomutase, chloroplastic* | UPI0000131858 |
|  | 51953_0_2 | *psb28* | *Photosystem II reaction center PSB28 protein, chloroplastic* | UPI00004018F2 |
|  | 45131_0_2 | *At1g34750* | *Probable protein phosphatase 2C 10* | UPI0000048333 |
|  | 52389_0_2 | *EDR2L* | *Protein ENHANCED DISEASE RESISTANCE 2-like* | UPI00000A5924 |
|  | 56792_3_5 | *FRS8* | *Protein FAR1-RELATED SEQUENCE 8* | UPI00017390ED |
|  | 51709_1_1 | *SGS3* | *Protein SUPPRESSOR OF GENE SILENCING 3* | UPI0001566472 |
|  | 15786_0_1 | *THF1* | *Protein THYLAKOID FORMATION 1, chloroplastic* | UPI00000A76CF |
|  | 56455_2_2 | *EMB1444* | *Transcription factor EMB1444* | UPI00005DBF20 |
|  | 41958_0_3 | *MYB3* | *Transcription factor MYB3* | UPI00000ABF81 |
|  | 30892_0_2 | *TMN3* | *Transmembrane 9 superfamily member 3* | UPI00000A85BB |
|  | 28120_0_1 | *At5g07050* | *WAT1-related protein At5g07050* | UPI000009ED6B |
| **Cluster 11** | 11446_0_2 | *CYN* | *Cyanate hydratase* | UPI0001945D7E |
|  | 49273_0_2 | *tsf* | *Protein TWIN SISTER of FT* | UPI0000694F2E |
|  | 48463_2_1 | *MORF8* | *Multiple organellar RNA editing factor 8, chloroplastic/mitochondrial* | UPI0000137C2A |
|  | 43393_0_1 | *RPS13* | *30S ribosomal protein S13, chloroplastic* | UPI0000134887 |
|  | 16890_0_1 | *OEP163* | *Outer envelope pore protein 16-3, chloroplastic/mitochondrial* | UPI00000A2CE4 |
|  | 83905_0_1 | *EXL4* | *GDSL esterase/lipase EXL4* | UPI00005DC03C |
|  | 52512_1_6 | *RPS5* | *30S ribosomal protein S5, chloroplastic* | UPI000009EA0F |
|  | 37784_0_2 | *FRO1* | *NADH dehydrogenase [ubiquinone] iron-sulfur protein 4, mitochondrial* | UPI0000135064 |
|  | 41501_0_2 | *CYB5* | *cytochrome b-5* | UPI000000C0F7 |
|  | 28584_0_2 | *SMAP1* | *Small acidic protein 1* | UPI00001288B4 |
|  | 39301_0_1 | *Chm* | *DNA mismatch repair protein MSH1, mitochondrial* | UPI00000A83FC |
|  | 52880_0_3 | *matK* | *Maturase K* | UPI0000029C5A |

Continued

| **Cluster** | **Unigene ID** | **Gene symbol** | **Gene name** | **Uniprot_Entry** |
| --- | --- | --- | --- | --- |
| **Cluster 5** | 19115_0_2 | *DHNAT1* | *1,4-dihydroxy-2-naphthoyl-CoA thioesterase 1* | UPI00000A78D7 |
|  | 56442_0_1 | *KAS1* | *3-oxoacyl-[acyl-carrier-protein] synthase I, chloroplastic* | UPI0000001184 |
|  | 38437_0_1 | *rpl32* | *50S ribosomal protein L32, chloroplastic* | UPI00005DC14E |
|  | 52586_0_2 | *RPL31* | *60S ribosomal protein L31-3* | UPI0000133F1D |
|  | 55990_1_3 | *dis2* | *Actin-related protein 2/3 complex subunit 2A* | UPI0000131FAB |
|  | 46437_0_1 | *ACA7* | *Alpha carbonic anhydrase 7* | UPI00000A870D |
|  | 42053_0_1 | *TIP2-1* | *Aquaporin TIP2-1* | UPI000009EC52 |
|  | 29675_0_2 | *ttcA* | *AT-rich interactive domain-containing protein 2* | UPI00017BE8F4 |
|  | 51142_3_2 | *AUX1* | *Auxin transporter protein 1* | UPI0000036314 |
|  | 45011_0_3 | *D27* | *Beta-carotene isomerase D27, chloroplastic* | UPI0001B07C18 |
|  | 50319_0_1 | *CIPK2* | *CBL-interacting serine/threonine-protein kinase 2* | UPI00001B3939 |
|  | 12577_0_1 | *COX2* | *Cytochrome c oxidase subunit 2* | UPI000012800C |
|  | 53413_1_3 | *CYP76C1* | *Cytochrome P450 76C1* | UPI00000014D6 |
|  | 53643_1_1 | *At5g07800* | *Flavin-containing monooxygenase FMO GS-OX-like 9* | UPI000002A51B |
|  | 44141_0_1 | *GSTT3* | *Glutathione S-transferase T3* | UPI00000A8F76 |
|  | 49033_1_2 | *GSTU17* | *Glutathione S-transferase U17* | UPI0000048BE6 |
|  | 28258_0_2 | *At2g30105* | *LRR repeats and ubiquitin-like domain-containing protein At2g30105* | UPI00000A053E |
|  | 33006_0_2 | *MLO8* | *MLO-like protein 8* | UPI000186C985 |
|  | 57085_0_1 | *LTP1* | *Non-specific lipid-transfer protein 1* | UPI000000BE3A |
|  | 54883_1_1 | *PLP6* | *Patatin-like protein 6* | UPI000013022A |
|  | 22924_0_1 | *ycf3* | *Photosystem I assembly protein Ycf3* | UPI00000AC68D |
|  | 49188_0_1 | *PSY* | *Phytoene synthase, chloroplastic* | UPI0000132848 |
|  | 50702_0_2 | *QRT3* | *Polygalacturonase QRT3* | UPI00000A903A |
|  | 35085_0_1 | *AAE1* | *Probable acyl-activating enzyme 1, peroxisomal* | UPI0000162E3C |
|  | 29665_0_1 | *PRP4* | *Proline-rich protein 4* | UPI00000AC1BE |
|  | 26452_0_1 | *AGO1* | *Protein argonaute 1* | UPI0000001AF9 |
|  | 42681_1_1 | *menD* | *Protein PHYLLO, chloroplastic* | UPI000019701E |
|  | 12262_0_2 | *TIC20-V* | *Protein TIC 20-v, chloroplastic* | UPI00001BA512 |
|  | 34991_0_1 | *At2g20870* | *Putative cell wall protein* | UPI00000AC0F7 |
|  | 35254_0_1 | *rbcL* | *Ribulose bisphosphate carboxylase large chain* | UPI0000052A0C |
|  | 39259_0_2 | *WHY1* | *Single-stranded DNA-binding protein WHY1, chloroplastic* | UPI0000133378 |
|  | 30582_0_2 | *At4g02530* | *Thylakoid lumenal 16.5 kDa protein, chloroplastic* | UPI00000A4B02 |
|  | 49331_0_1 | *TPR4* | *Topless-related protein 4* | UPI000009E653 |
|  | 56964_0_3 | *UBC30* | *Ubiquitin-conjugating enzyme E2 30* | UPI00001633E8 |
|  | 34896_0_3 | *At5g01610* | *Uncharacterized protein At5g01610* | UPI0000196CD4 |
|  | 31986_0_1 | *At5g48480* | *Uncharacterized protein At5g48480* | UPI000005EA56 |
|  | 49016_2_1 | *At1g09380* | *WAT1-related protein At1g09380* | UPI00000A1B3F |
|  | 17460_0_3 | *MSI2* | *WD-40 repeat-containing protein MSI2* | UPI00000A4702 |
|  | 31508_0_4 | *MSI4* | *WD-40 repeat-containing protein MSI4* | UPI000000C6DC |
|  | 33488_0_3 | *YLMG2* | *YlmG homolog protein 2, chloroplastic* | UPI0000000E30 |
| **Cluster 15** | 28584_0_1 | *SMAP1* | *Small acidic protein 1* | UPI00000A83FC |
|  | 83905_0_2 | *EXL4* | *GDSL esterase/lipase EXL4* | UPI000009EA0F |
|  | 76698_0_1 | *psbA* | *Photosystem II protein D1* | UPI00003A92DB |
|  | 48127_0_1 | *GGAT2* | *Glutamate--glyoxylate aminotransferase 2* | UPI00000AC411 |
|  | 17119_0_1 | *unassigned* | *Desiccation protectant protein Lea14 homolog* | UPI000012E375 |
